# SCD1 inhibition promotes lipid peroxidation and enhances anti-PD-1 response in triple-negative breast cancer

**DOI:** 10.3389/fimmu.2026.1843935

**Published:** 2026-10-12

**Authors:** Quentin Herrera Garfia, Aurélien Azam, Eric Bonneil, Laetitia Montero-Ruiz, Mohsen Momeni, Mohammad Farran, Robin Jehay, Nor Eddine Sounni

**Affiliations:** 1 Cancer Metabolism and Tumor Microenvironment Laboratory, Interdisciplinary Group for Applied Genoproteomics, University of Liège, Liège, Belgium; 2 Institute for Research in Immunology and Cancer (IRIC), University of Montréal, Montréal, QC, Canada; 3 Laboratory of Tumor and Development Biology Laboratory, Interdisciplinary Group for Applied Genoproteomics, University of Liège, Liège, Belgium

**Keywords:** anti-PD1, ferroptosis, immunotherapy, MIF, SCD1, TNBC, ferroptosis inducers

## Abstract

**Background:**

Triple-negative breast cancer (TNBC) is a heterogeneous disease that often exhibits a relatively immunologically “cold” tumor microenvironment, which can limit the efficacy of immune checkpoint inhibitors (ICIs). Alterations in lipid metabolism and lipid peroxidation may influence tumor–immune interactions and represent potential determinants of response to immune checkpoint blockade.

**Methods:**

Human TNBC cell lines (MDA-MB-231, BT-549) were treated with ferroptosis inducers (FINs), including RSL3 and SCD1 inhibitor (SCD1i), and cell death was assessed. Proteomic profiling of cell lysates and conditioned media was performed, and IL-8 and MIF secretion was quantified by ELISA. Splenocytes were incubated with conditioned media to assess immune activation via flow cytometry. *In vivo* efficacy of SCD1i, PD-1 blockade, and their combination was evaluated using the E0771 murine TNBC model.

**Results:**

SCD1i and RSL3 treatment induced the release of inflammatory mediators, including IL-8 and MIF, as confirmed by ELISA. Conditioned media from treated TNBC cells enhanced immune activation in splenocytes, characterized by increased effector CD4^+^ and CD8^+^ T-cell populations and altered expression of immune checkpoint receptors. *In vivo*, SCD1 inhibition increased CD8+ T cell infiltration into the tumor microenvironment and increased PD-1 and LAG-3 expression in both CD4+ and CD8^+^ T cells. Notably, combined SCD1 inhibition and anti-PD-1 therapy resulted in greater tumor growth inhibition compared with either treatment alone.

**Conclusion:**

SCD1 inhibition promotes lipid peroxidation and release of inflammatory factors that enhance immune activation and sensitize TNBC to checkpoint blockade. Targeting the SCD1 represents a promising strategy to improve ICI efficacy in TNBC.

## 1 Introduction

Breast cancer is the most common type of cancer in women, and triple-negative breast cancer (TNBC), which does not express estrogen (ER), progesterone (PR), and human epidermal growth factor receptor-2 (HER2), accounts for 15-20% of cases and is the type of breast cancer with the poorest prognosis (1). The standard treatment for TNBC is chemotherapy, as this type of cancer does not respond to hormone and targeted therapies because it does not express ER, PR, and HER2. Notably, the immunogenicity of TNBC is heterogeneous, with only a subset of tumors exhibiting an immune-active tumor microenvironment characterized by prominent lymphocyte infiltration within the tumor microenvironment (2). This enhanced immunogenicity has been associated with elevated immune-related gene expression signatures and increased levels of tumor-infiltrating lymphocytes (TILs) observed in TNBC tumors compared with other breast cancer subtypes (3). However, TNBC cells also express high levels of programmed cell death-ligand 1 (PD-L1), making the microenvironment highly immunosuppressive (4). Immunotherapy has therefore emerged as an important therapeutic option for TNBC. Atezolizumab, an anti-PD-L1 antibody, received accelerated FDA approval in 2019 for PD-L1-positive metastatic TNBC; this indication was subsequently withdrawn in the United States. Pembrolizumab, an anti-PD-1 antibody, was approved by the FDA in 2020 for PD-L1-positive unresectable or metastatic TNBC and in 2021 for high-risk early-stage TNBC in combination with chemotherapy, an indication that was not restricted to PD-L1-high disease. Nevertheless, the clinical benefit of immune checkpoint blockade remains heterogeneous, highlighting the need to identify biomarkers and therapeutic strategies that can improve treatment response (5, 6).

Ferroptosis is a non-apoptotic cell death mechanism linked to an accumulation of peroxidized lipids, mainly polyunsaturated fatty acids (PUFAs), in the cell membrane, leading to its rupture (7). Lipid peroxidation is strongly linked to iron and reactive oxygen species (ROS) levels in the cell. To inhibit ferroptosis, cells have developed several mechanisms, including lipid detoxification via the enzyme GPX4, using glutathione. Several ferroptosis inducers have therefore been developed to inhibit this detoxification, for example erastin, an inhibitor of SLC7A11, the transporter of cysteine in the cell, which is essential for the formation of glutathione, and RAS selective lethal 3 (RSL3), an inhibitor of GPX4 (8). Ferroptosis resistance has been recognized as a key contributor to drug resistance in breast cancer, particularly in response to hormone therapy, through mechanisms involving the GPX4-dependent pathway (9, 10). In addition, alterations in lipid metabolism have emerged as important drivers of therapeutic resistance through enhanced *de novo* lipid synthesis (11). More recent studies have also identified a GPX4-independent mechanism of ferroptosis resistance mediated by stearoyl-CoA desaturase-1 (SCD1), which shifts the balance of PUFAs toward monounsaturated fatty acids (MUFAs), thereby reducing lipid peroxidation and ferroptotic sensitivity (12). Importantly, ferroptotic cancer cells have been shown to stimulate antitumor immune responses through the release of damage-associated molecular patterns and activation of dendritic cells, suggesting a potential link between ferroptosis and cancer immunotherapy (13). However, cancer cells frequently develop ferroptosis resistance through metabolic adaptations in lipid metabolism, including pathways regulated by SCD1, which modulates the balance between polyunsaturated and monounsaturated fatty acids and reduces susceptibility to lipid peroxidation (12). Targeting SCD1 may therefore represent a potential strategy to overcome ferroptosis resistance while simultaneously enhancing antitumor immune responses in TNBC.

Apoptotic cell death communicates with immune cells via “find-me” and “eat-me” signals (14). By contrast, the immunological consequences of alternative cell death modalities, including ferroptosis, remain poorly understood. Here, we found that ferroptotic tumor cells release a distinct secretome including oxidized lipid species, macrophage migration inhibitory factor (MIF), and IL-8. Conditioned medium from TNBC cells undergoing SCD1 inhibition enhanced effector CD4^+^ and CD8^+^ T cell activation in splenic cultures and reduced immune checkpoint expression markers. *In vivo*, SCD1 inhibition increased T cell infiltration into the tumor microenvironment but was accompanied by compensatory upregulation of PD-1 and LAG-3 expression in CD4+ and CD8+ T cells, suggesting a compensatory checkpoint response. Thus, SCD1 inhibition enhances T cell infiltration while simultaneously inducing checkpoint upregulation, highlighting a dynamic balance between immunostimulatory and immunosuppressive signals. Accordingly, combining SCD1 inhibition with anti-PD-1 immunotherapy enhanced T cell–mediated anti-tumor responses and produced greater tumor growth inhibition than either treatment alone. Collectively, these findings suggest that SCD1 inhibition promotes lipid peroxidation and a ferroptosis-associated secretory program that stimulates antitumor immunity while simultaneously eliciting compensatory checkpoint signaling. Targeting SCD1 in combination with immune checkpoint blockade may therefore represent a promising strategy to enhance immunotherapy efficacy in TNBC.

## 2 Materials and methods

### 2.1 Cell culture

Human breast cancer cells (MDA-MB-231, BT-549, SK-BR-3, HCC-1954, MCF-7, T47-D) and the murine mammary cancer cell line, E0771, were purchased from American Type Culture Collection (ATCC) (Manassas, Virginia, USA). Cell line authentication for interspecies contamination was performed by Leibniz-Institute, Deutsche Sammlung von Mikroorganismen und Zellkulturen (DSMZ) (Braunschweig, Germany). Cell lines were tested by MycoALert Kit 139 (Lonza – Basel, Switzerland) to ensure that they were Mycoplasma-free before *in vitro* and *in vivo* experiments. MCF7, SK-BR-3, BT-549 and MDA-MB-231 cells were grown in Dulbecco’s modified Eagles’s medium (DMEM) (10938025, Thermofisher, Waltham, Massachusetts, USA), supplemented with 10% fetal bovine serum (FBS) (10270-106, ThermoFisher), L-glutamine (2 mM) (25030-24, ThermoFisher) and 1% Penicillin-Streptomycin (15140-122, ThermoFisher) at 37 °C, in a 5% CO2 humid atmosphere. HCC1954, T47-D and E0771 were grown in Roswell Park Memorial Institute (RPMI) 1640 medium (21875-034, Thermofisher), supplemented with 10% FBS, and 1% Penicillin-Streptomycin at 37 °C, in a 5% CO_2_ humid atmosphere. Treatments.

To induce ferroptosis, cells were treated with erastin (HY-15763, MedChemExpress, New Jersey, USA), RSL3 (HY-100218A, MedChemExpress) or an inhibitor of Stearoyl-CoA desaturase 1 (SCD1i) (T4515, TargetMol Chemicals Inc, Massachusetts, USA). To inhibit ferroptosis, Deferoxamine (Def) (50µM) (HY-B1625, MedChemExpress) was administered 2 h prior to ferroptosis inducers (FINs) treatment. Apoptosis was induced using Staurosporine (STS) (100nM) (HY-15141, MedChemExpress), and the cells were treated with Z-VAD-FMK (50µM) (HY-16658B, MedChemExpress) 2 h prior to STS treatment to inhibit apoptosis. All drugs were diluted in dimethylsulfoxide (DMSO), with a final DMSO concentration below 0.2% in all vitro experiments.

### 2.2 Cell proliferation assays and IC_50_ calculations

IC50 of various FINs, erastin, RSL3 and SCD1i, were calculated by proliferation assays using the Incucyte SX5 (Sartorius, Goettingen, Germany). Cells (5000/well) were seeded in triplicate per condition in a 96-well plate and confluency was measured at day 1 before treatment and every day until 3 days after treatment. SPY650-DNA (1/1.000) (SC501, TebuBio, Le Perray-en-Yvelines, France) and SYTOX™ Green Nucleic Acid Stain (20nM) (S7020, Invitrogen, Carlsbad, California, USA) were added to the culture medium 2 h before the first scan to label total cell nuclei and dead cells, respectively.

### 2.3 Determination of lipid peroxidation levels

BODIPY™ 581/591 C11 is a fluorescent probe designed to detect lipid peroxidation in living cells. Its lipophilicity allows it to incorporate into lipid-rich regions, such as cell membranes. Lipid peroxidation causes a shift in the fluorescent emission peak of the probe from approximately 590 nm (red) to approximately 510 nm (green). Breast cancer cells were treated with FINs for 6 and 24 h, then washed with PBS, trypsinized, and incubated with the fluorescent probe BODIPY™ 581/591 C11 (D3861, ThermoFisher) for 30 minutes at 37 °C and 5% CO_2_. The cells were then analyzed by flow cytometry to observe the shift toward green of the probe.

### 2.4 Proteomic analysis

MDA-MB-231 cells were treated in Petri dishes with SCD1i (15 µM), or RSL3 (1 µM) for 6 or 24 hours. SCD1i and RSL3 were diluted in DMEM medium without FBS. At the end of treatment, the conditioned media at 24 h, as well as the cell pellets at 6 and 24 hours, were collected and stored at -80 °C until analysis. Samples were reconstituted in 50 mM ammonium bicarbonate with 10 mM TCEP [Tris(2-carboxyethyl) phosphine hydrochloride; Thermo Fisher Scientific], and vortexed for 1 h at 37 °C. Chloroacetamide (Sigma-Aldrich) was added for alkylation to a final concentration of 55 mM. Samples were vortexed for another hour at 37 °C. One microgram of trypsin was added, and digestion was performed for 8 h at 37 °C. Samples were dried down and solubilized in 4% formic acid (FA). Peptides were loaded and separated on a home-made reversed-phase column (150-μm i.d. by 200 mm) with a 56-min gradient from 10 to 30% ACN-0.2% FA and a 600-nl/min flow rate on an Easy nLC-1000 connected to an Orbitrap Fusion (Thermo Fisher Scientific, San Jose, CA). Each full MS spectrum acquired at a resolution of 120,000 was followed by tandem-MS (MS-MS) spectra acquisition on the most abundant multiply charged precursor ions for a maximum of 3s. Tandem-MS experiments were performed using collision-induced dissociation (CID) at a collision energy of 30%. The data were processed using PEAKS X Pro (Bioinformatics Solutions, Waterloo, ON) and a Uniprot human database (20366 entries). Mass tolerances on precursor and fragment ions were 10 ppm and 0.3 Da, respectively. Fixed modification was carbamidomethyl (C). Variable selected posttranslational modifications were oxidation (M), deamidation (NQ), phosphorylation (STY) along acetylation (N-ter). Two sample t-test were performed to get log2 of the fold change and –log of p-values. The mass spectrometry proteomics data have been deposited to the ProteomeXchange Consortium via the PRIDE partner repository with the dataset identifier PXD082708 and 10.6019/PXD082708.

### 2.5 Enzyme-linked immunosorbent assay

MDA-MB-231 (3.10^5^) and E0771 (4.10^5^) cells were plated in 6 well-plates with DMEM 10% FBS or RPMI 1640 10% FBS respectively. 24 hours after seeding, the cells were washed with PBS and treated as described in the “Treatment” section, in 1mL medium without FBS. The conditioned medium of MDA-MB-231 and E0771 cells were collected after 24 hours of treatment. MIF levels were measured in the conditioned medium of both cell types, and IL-8 levels were measured only in the conditioned medium of human cells. MIF and IL-8 levels in the conditioned media were measured following the instructions for the Mouse MIF DuoSet (DY1978, R&D Systems, Minneapolis, Minnesota), Human MIF DuoSet (DY289, R&D Systems), and Human IL-8/CXCL8 DuoSet ELISA (DY208, R&D Systems) kits.

### 2.6 Splenocyte harvesting and culture in conditioned medium

Female C57Bl/6 organ donor spleens were harvested and placed in of 12-well plates containing 1 ml of PBS, on ice, in order to isolate the splenocytes. The tissues were crushed using the flat plunger of a 10 ml syringe and, using an 18G needle mounted on a 10 ml syringe, the cells were dissociated by up and down movement to obtain a cell suspension. This cell suspension is then filtered through a 70µm filter and centrifuged at 300g for 10 min at 4 °C. The supernatant is removed, and the cells are resuspended in 5 mL of 1x RBC Lysis Buffer (2701510, Sony Biotechnology Inc., California, USA) and incubated for 5 min at room temperature. 5 mL of PBS is then added to inactivate the lysis. The cells are counted using the Corning^®^ Cell Counter (Corning, 6749), and viability is analyzed using Trypan Blue. Finally, 3x10^6^ cells were deposited in the well of a 6-well plate in 2 mL of conditioned medium from E0771 cells treated for 24 hours with FINs. The splenocytes were incubated overnight at 37 °C, in a 5% CO2 humid atmosphere. The splenocytes were then harvested and prepared for flow cytometry analysis.

### 2.7 Syngeneic E0771 TNBC model and *in vivo* treatment with SCD1 inhibitor and anti-PD-1 antibody

E0771 cells (5 × 10^5^) were injected subcutaneously (s.c.) into both flanks of female C57BL/6 mice (6–8 weeks old; Charles River) to establish a syngeneic model of TNBC. The mice were randomly assigned to the different treatment groups before tumor cell injection. Tumor growth was monitored every 2 to 3 days, and tumor volume was calculated using the following formula: (L x l²) x 0.4, L and l represent the tumor length and width, respectively. When tumors reached 70–100 mm³, mice were treated daily by oral gavage with an SCD1 inhibitor T4515, (TargetMol) at 40 mg/kg or with vehicle control (placebo). T4515 was dissolved in DMSO (vehicle) at 40 mg/mL as a stock solution, then diluted in the vehicle formulation and sonicated prior to administration. The final vehicle formulation used for T4515 consisted of 10% DMSO in PBS. In earlier *in vivo* experiments, the SCD1 inhibitor Abcam #Ab142089 was used and formulated in 2.5% DMSO, 0.5% methylcellulose, and 0.25% Tween 80 in water. For PD-1 blockade, mice received intraperitoneal (i.p.) injections of anti-mouse PD-1 antibody (clone RMP1-14; MedChemExpress, HY-P99144) at 10 mg/kg (0.2 mg per injection per mouse), administered twice weekly on days 1 and 3. Control mice received an IgG isotype control (Rat IgG2a kappa, Isotype Control) (MedChem Express, HY-P990679). For combination therapy (SCD1 inhibitor + anti-PD-1), control (placebo) mice received both corresponding controls (vehicle for SCD1 inhibitor and IgG isotype for anti-PD-1). At the end of the experiment, mice were euthanized by cervical dislocation, and tumors were harvested for flow cytometry and immunohistochemical analysis. Tumors were cut in half, with one half fixed and embedded in paraformaldehyde (PFA) and the other half processed for flow cytometry. All animal procedures were performed in accordance with the guidelines of Federation of European Laboratory Animal Science Associations (FELASA) in an accredited institutional animal facility, and under the approved animal protocol #2458 at the University of Liège, Belgium.

### 2.8 Immunohistochemistry

The half tumors resected from mice were fixed in PFA 10%immediately after resection, dehydrated in ethanol, and embedded in paraffin. The paraffin blocks were cut into 5µm slices for 4-Hydroxynonenal (4-HNE) labeling. The slides were deparaffinized and then autoclaved in citrate buffer. Endogenous peroxidases were blocked with H_2_O_2_, followed by blocking with an animal-free blocking solution (15019, Cell Signaling). The slides were incubated for 1 hour with the primary antibody 4-HNE Antibody (MAB3249, R&D Systems, 1:1000). After rinsing, the slides were incubated for 30 minutes in the Dako EnVision+ System- HRP Labelled Polymer Anti-mouse (Dako K4001). The slides were reveled with SignalStain^®^ DAB Substrate Kit (#8059, Cell Signaling) for approximately 5 minutes. The slides were counterstained with hematoxylin and mounted with Entellan™ new (1.07961.0100, Sigma-Aldrich). Slides were scanned by Nanozoomer Digital Pathology (NDP) System (Hamammatsu - Shizuoka, Japan). Quantification of 4-HNE immunohistochemistry was performed by QuPath v3.2 (Open-source software for digital pathology image analysis, https://qupath.github.io) and following the website manual.

### 2.9 Tumor digestion

The two-halve of each tumor were pooled and digested together in a solution containing 3 mg/ml collagenase D (11088866001, Sigma-Aldrich, Missouri, USA) and 0.2 mg/ml DNase 1(11284932001, Sigma-Aldrich) diluted in Hanks′ Balanced Salt Solution (HBSS, H4641-100ML, Sigma-Aldrich) for approximately 1 hour at 37 °C. The cell suspension is then filtered through a 70 µm filter and centrifuged at 300 g for 10 min at 4 °C. The supernatant is removed, and the cells are resuspended in 5 mL of 1x RBC Lysis Buffer and incubated for 5 min at room temperature. 5 mL of PBS is then added to inactivate the lysis. The cells are counted using the Corning^®^ Cell Counter (Corning, 6749), and 5x10^6^ cells were collected from each sample for flow cytometry analysis.

### 2.10 Flow cytometry analysis

Splenocytes cultured in conditioned medium and cells derived from tumor digestion were labeled to study the immune system’s response to ferroptosis-induced tumor cell death. The cells were first labeled with Zombie-Nir (423105, BioLegend, Californie, USA), diluted in PBS, to study cell viability. The cells were then labeled with a CD45 (103151, BioLegend) marker for leukocytes, CD3 (100271, BioLegend) for T lymphocytes, and CD4 (1102410, Sony) and CD8a (1103750, Sony) to differentiate between helper and cytotoxic T lymphocytes, respectively. These two populations were differentiated into CD4+ or CD8+ effector (CD44+/CD62L-), memory (CD44+/CD62L+), and naïve (CD44-/CD62L+) T lymphocytes (CD44, 1115235, Sony) (CD62L, 1122320, Sony). Finally, the cells were also labeled with markers for CD69 (1122790, Sony), PD-1 (752299, BD Biosciences, New Jersey, USA), LAG-3 (125209, BioLegend), Tim-3 (1270105, Sony), CTLA-4 (1131570, Sony) and BTLA (755187, BD Biosciences). All antibodies were diluted in FACS Buffer.

### 2.11 Western blot analysis

Total protein was extracted using Cell Lysis Buffer (# 9803, Cell Signaling) (10x diluted in MilliQ water) supplemented with cOmplete™ Protease Inhibitor Cocktail (04693116001, Roche) and PhosSTOP™ phosphatase inhibitor (4906837001, Roche). Following 30 min incubation on ice with intermittent vortexing, cell lysates were clarified by centrifugation at 10,000 × g for 10 min at 4 °C, and supernatants were collected. Protein concentrations were determined using the DC™ protein assay kit (5000112, Bio-Rad) according to the manufacturer’s instructions, with absorbance measured at 750 nm. Equal amounts of protein (20 µg per lane) were resolved by SDS-PAGE on 15% polyacrylamide gels and electrophoretically transferred onto PVDF membranes (88518, ThermoFisher Scientific) at 100 V for 60 min at 4 °C in transfer buffer (25 mM Tris, 192 mM glycine, 20% methanol). Membranes were blocked with 5% non-fat dry milk in Tris-buffered saline containing 0.1% Tween-20 (TBS-T) for 2 h at room temperature (RT) and then incubated overnight at 4 °C with primary antibodies diluted in blocking buffer. The following primary antibodies were used: rabbit anti-MIF monoclonal antibody (ab187064, Abcam 1:1000) and mouse anti-GAPDH monoclonal antibody (MAB374, Chemicon International, 1:10000). After three washes with TBS-T (5 min each), membranes were incubated with HRP-conjugated secondary antibodies Goat anti-rabbit IgG (7074, Cell Signaling, 1:2000) or Horse anti-mouse IgG (7076, Cell Signaling, 1:2000) for 1h at RT. Protein bands were visualized using enhanced chemiluminescence Pierce™ ECL Western Blotting Substrate (32209, Thermo Fisher Scientific) and images were captured using a ChemiDoc imaging system (Bio-Rad). GAPDH served as a loading control.

### 2.12 MIF siRNA transfection

E0771 cells were seeded in a 6-well plate. The next day, the cells were transfected with MIF siRNA (L2-040022-01-0005, Horizon Discovery) or control siRNA (D2-001810-10-05, Horizon Discovery) in the presence of Lipofectamine RNAiMAX (13778100, Thermo Fisher Scientific). 24 hours after the first transfection, a second transfection is performed for 24h.

### 2.13 Statistical analysis

Statistical analyses of *in vitro* assays results were performed using one-way ANOVA or Kruskal–Wallis tests for comparisons involving more than two groups, and an unpaired Student’s *t*-test or Mann–Whitney *U* test for comparisons between two groups, as appropriate. For experiments in mice, two-way ANOVA was used for longitudinal tumor growth analyses, while one-way ANOVA or Kruskal–Wallis tests were used for comparisons involving more than two groups, and an unpaired Student’s *t*-test or Mann–Whitney *U* test for comparisons between two groups, as appropriate. The decision to use a parametric or nonparametric test was based on a Normality and Lognormality distribution, using Shapiro-Wilk normality test and Kolmogorov-Smirnov test. Outliers were identified using Grubbs’ test and excluded from the analysis where appropriate. Data are presented as mean ± SD unless otherwise indicated. Statistical analyses and graphical representations were generated using GraphPad Prism 9.0 (Dotmatics, Boston, USA).

## 3 Results

### 3.1 Differential sensitivity of breast cancer subtypes to ferroptosis inducers and lipid metabolism inhibitor

To evaluate ferroptosis susceptibility across breast cancer subtypes, breast cancer cells were treated with increasing concentrations of FINs, including the GPX4 inhibitor RSL3, the SLC7A11/xCT inhibitor erastin, and the SCD1 inhibitor (SCD1i) (**Figure 1A**; **Supplementary Figure 1**). IC_50_ values were determined for classical ferroptosis inducers RSL3 and erastin, as well as for the lipid metabolism inhibitor SCD1i, in a panel of cell lines representing triple-negative (BT-549, MDA-MB-231), luminal (MCF-7, T47-D), and HER2-positive (HCC1954, SK-BR-3) breast cancer subtypes. Across all cell lines, RSL3 displayed the highest potency, with IC_50_ values in the submicromolar range (0.06–1.92 µM) (**Figure 1**; **Table 1**). The HER2-positive cell line HCC1954 was particularly sensitive (IC_50_ = 0.06 µM), whereas MCF-7 cells exhibited the highest IC_50_ within this group (1.92 µM). In comparison, erastin showed greater variability in efficacy, with IC_50_ values ranging from 0.76 to 13.5 µM (**Supplementary Figure 1**; **Table 1**). Triple-negative cell lines (BT-549 and MDA-MB-231) were more sensitive to erastin (0.76 µM and 2.12 µM, respectively), whereas luminal MCF-7 and HER2-positive SK-BR-3 cells displayed relative resistance (13.5 µM and 11.97 µM, respectively) (**Supplementary Figure 1**; **Table 1**).

**Figure 1:**
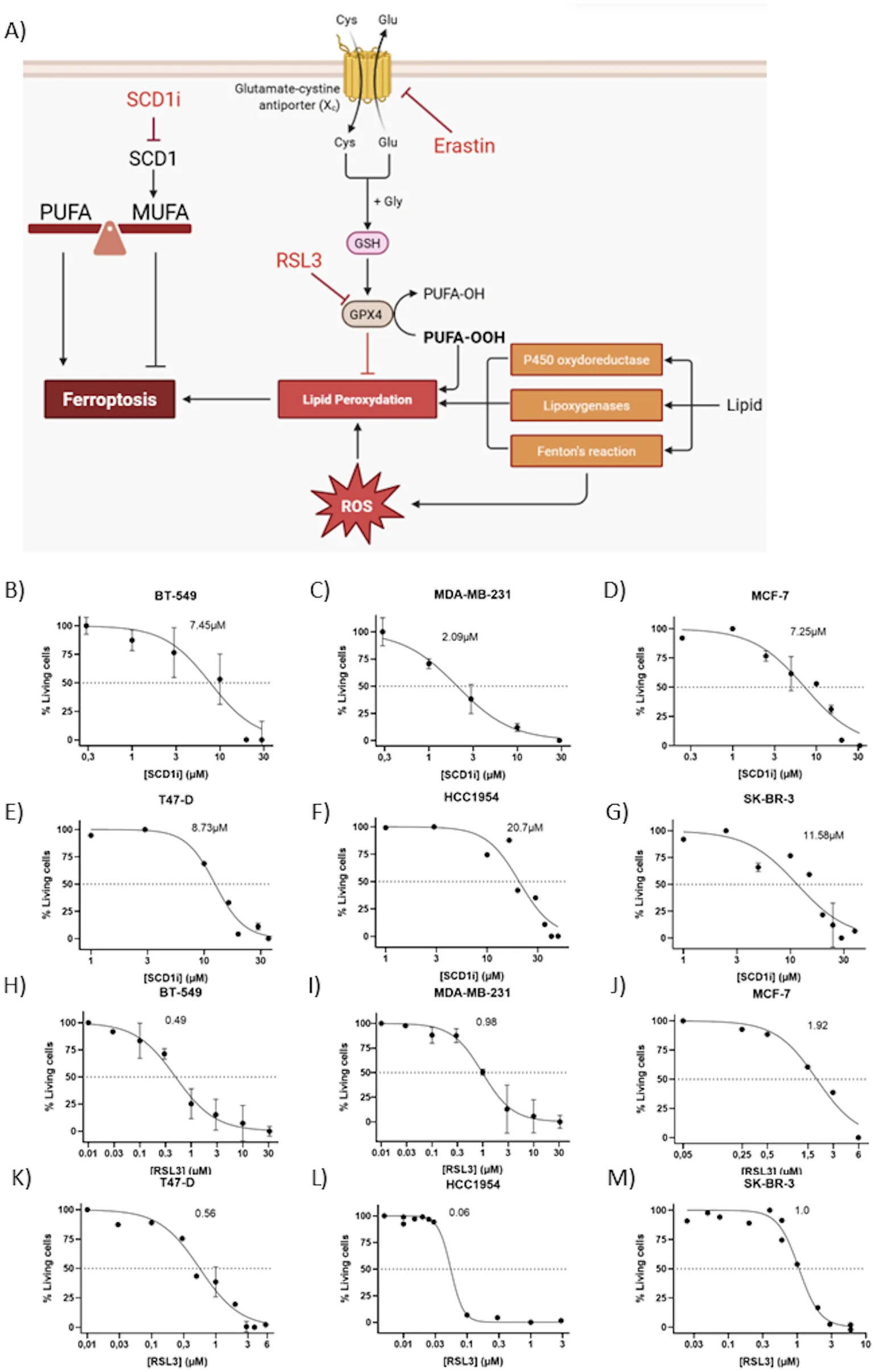
Sensitivity of breast cancer cells to SCD1 inhibition and RSL3-induced ferroptosis. **(A)** Ferroptosis induction in breast cancer cell lines was achieved through multiple pathways: (i) inhibition of GPX4, the rate-limiting enzyme responsible for detoxifying lipid peroxides, using RSL3; (ii) blockade of the glutamate–cystine antiporter system Xc^-^ by erastin; and iii) inhibition of lipid desaturation via SCD1 inhibitor (SCD1i). Breast cancer cells were treated with increasing concentrations of the SCD1i **(B–G)** or the GPX4 inhibitor RSL3 **(H–M)**. Cell proliferation was monitored by IncuCyte live-cell imaging, and growth inhibition was determined based on the number of viable cells (n = 3). *RSL3, RAS-selective lethal 3; SCD1i, stearoyl-CoA desaturase 1 inhibitor*. **Figure 1A** was created using BioRender and adapted under the appropriate license at GIGA, University of Liège.

**Table 1:** Breast cancer cell response to ferroptosis inducers *in vitro* (IC50).

BC subtype	ER-/PR-/HER2-		ER+/PR+/HER2-		ER+/PR+/HER2+	
Cell line FIN	BT-549	MDA-MB-231	MCF-7	T47-D	HCC1954	SK-BR-3
SCD1i	7,45 µM	2,09 µM	7,25 µM	8,73 µM	20,7 µM	16,5 µM
RSL3	0,49 µM	0,98 µM	1,92 µM	0,56 µM	0,06 µM	1,0 µM
Erastin	0,76 µM	2,12 µM	13,5 µM	2,06 µM	2,44 µM	11,97 µM

Using the IncuCyte SX5 live-cell imaging system, we tested the sensitivity of six human breast cancer cell lines to four ferroptosis inducers (FINs). The SCD1 inhibitor (SCD1i) was compared with two well-established ferroptosis inducers, RSL3 and erastin. IC_50_ values were determined based on cell confluence measurements after 24 h of treatment.

In contrast to classical ferroptosis inducers, SCD1i exhibited reduced potency overall (**Figure 1**; **Table 1**). SCD1 inhibition resulted in intermediate IC_50_ values (2.09–20.7 µM), with MDA-MB-231 cells showing the greatest sensitivity (2.09 µM), while HER2-positive cell lines (HCC1954 and SK-BR-3) were less responsive (20.7 µM and 16.5 µM, respectively).

Stratification by subtype revealed distinct response patterns. Luminal cell lines demonstrated greater resistance, particularly to erastin. HER2-positive models showed heterogeneous responses, characterized by pronounced sensitivity to RSL3 (notably HCC1954) but reduced susceptibility to lipid metabolism inhibitor. In contrast, triple-negative breast cancer cells exhibited relatively high sensitivity to both ferroptosis inducers and SCD1 inhibition, suggesting increased dependence on lipid peroxidation pathways.

### 3.2 Lipid peroxidation is differentially induced across breast cancer subtypes

To determine whether the observed differences in drug sensitivity were associated with ferroptotic lipid damage, lipid peroxidation levels were quantified by flow cytometry following treatment with ferroptosis inducers and lipid metabolism inhibitor (**Figure 2**). Cells were first gated to exclude debris and doublets, ensuring analysis of viable single-cell populations (**Figure 2A**). Lipid peroxidation was then assessed by measuring the shift in C11-BODIPY fluorescence intensity relative to untreated controls (**Figure 2**) and quantified as mean fluorescence intensity (MFI) (**Supplementary Figure 2**). The specificity of lipid peroxidation was confirmed by pre-incubating cells with the lipid-soluble antioxidant Trolox (**Supplementary Figures 2A, B, E**). Across all cell lines, treatment with RSL3 induced a clear rightward shift in fluorescence intensity, consistent with increased lipid peroxidation. This effect was most pronounced in triple-negative (BT-549, MDA-MB-231) and HER2-positive HCC1954 cells, which exhibited substantial increases in oxidized lipid signal compared to controls (**Figures 2B, C**). In contrast, luminal cell lines (MCF-7 and T47-D) displayed more modest shifts, indicating lower levels of lipid peroxidation upon treatment (**Figure 2D**). In line with its weaker cytotoxic effect, SCD1 inhibition resulted in a detectable rightward shift in triple-negative cell lines, particularly MDA-MB-231, suggesting that disruption of MUFA synthesis promotes lipid peroxidation in these cells. Notably, the magnitude of lipid peroxidation closely paralleled the IC_50_ data, with cells most sensitive to RSL3 exhibiting the strongest accumulation of lipid peroxides, supporting a ferroptosis-dependent mechanism of cell death. Conversely, more resistant cell lines displayed attenuated lipid peroxidation responses. Nevertheless, SCD1 inhibition induced a detectable rightward shift in C11-BODIPY fluorescence in triple-negative cell lines (**Figure 2B**), particularly shift MDA-MB-231 and BT-549 cells at 6 h of treatment, as well as in HCC1954 and MCF7 cells after 24 h (**Supplementary Figure 2**), indicating that disruption of MUFA synthesis promotes lipid peroxide accumulation in these cells.

**Figure 2:**
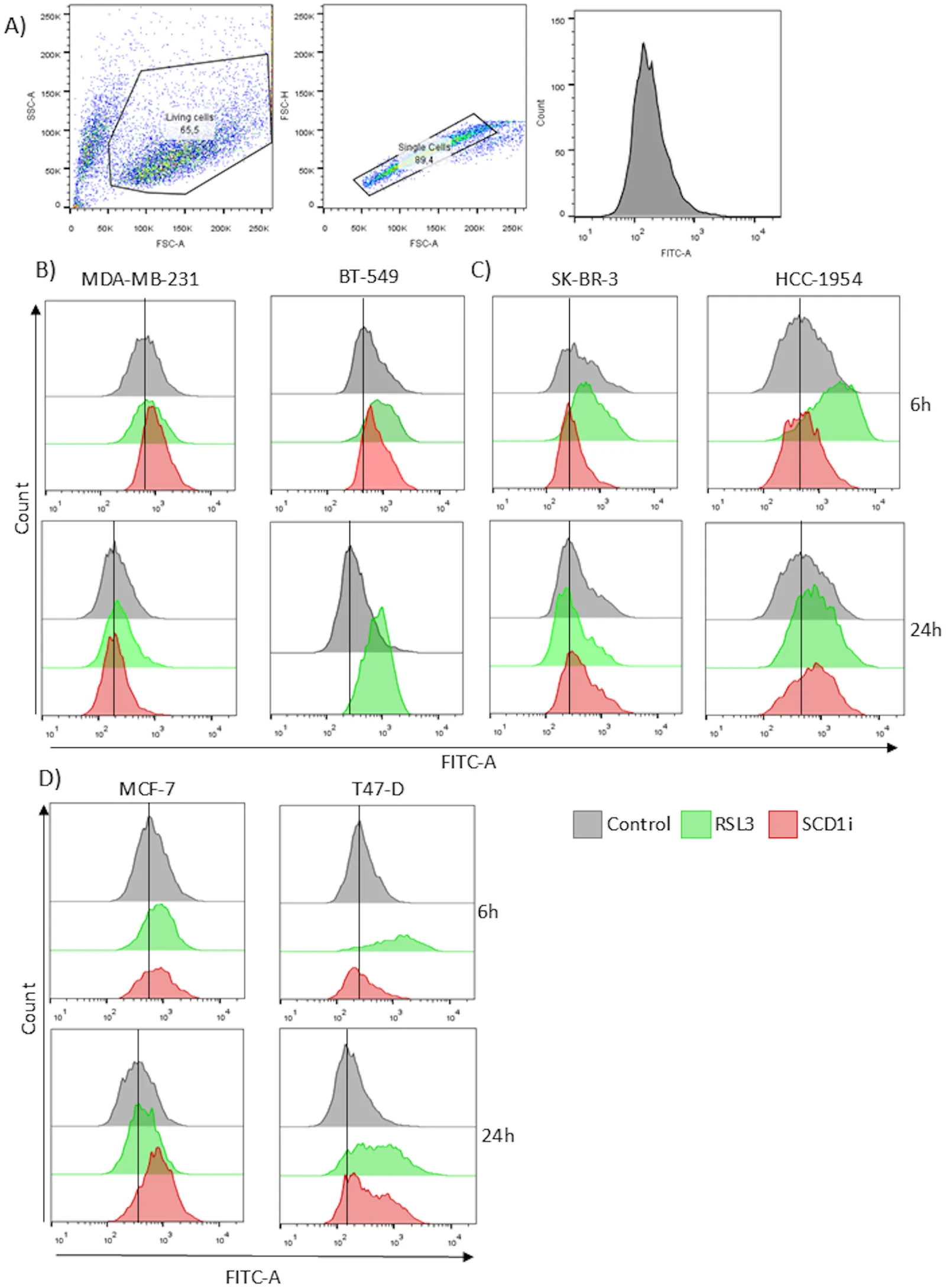
SCD1 inhibition increases lipid peroxidation in breast cancer cells. Lipid peroxidation was assessed by flow cytometry using the fluorescent lipid peroxide probe BODIPY™ 581/591 C11. **(A)** Gating strategy: live cells were first selected, followed by doublet exclusion, and fluorescence shifts were analyzed in the FITC channel. B–D) Lipid peroxidation was measured in triple-negative breast cancer (TNBC) cell lines (MDA-MB-231 and BT-549) **(B)**, HER2-positive cell lines (SK-BR-3 and HCC1954) **(C)**, and hormone receptor-positive cell lines (MCF-7 and T47D) **(D)** following treatment with DMSO (vehicle control), RSL3 (1 μM), or the SCD1 inhibitor (SCD1i, 20 μM) for 6 or 24 h. DMSO served as the negative control, whereas RSL3 was used as a positive control for ferroptosis induction. *DMSO, dimethylsulfoxide; RSL3, RAS-selective lethal 3; SCD1i, stearoyl-CoA desaturase 1 inhibitor*.

### 3.3 Ferroptosis-inducing treatments reshape the secretome and enrich for immune-related factors in TNBC cells

To investigate whether ferroptosis-inducing treatments modulate the tumor secretome, proteomic analysis was performed on conditioned media from MDA-MB-231 cells treated with SCD1i or RSL3. Cells were exposed to each compound for 6 h and 24 h, and both conditioned media and cell pellets were analyzed (**Figures 3A–F**). Across all conditions, a substantial number of proteins were differentially regulated (**Supplementary Figure 3**), indicating a broad remodeling of the extracellular proteome in response to ferroptotic stress. Comparative analysis revealed both treatment-specific and shared changes in protein secretion. Venn diagram analysis of upregulated proteins identified a limited but consistent subset of factors commonly induced by ferroptosis-inducing treatments (**Figures 3A–C**). Notably, six immune-related proteins were consistently upregulated across all conditions, suggesting a conserved secretory response to ferroptosis. Among these shared proteins, several are associated with immune regulation and inflammatory signaling, including LAT2, HSPB11, PXDN, LAIR1, IL-8, and MIF (**Table 2**). The enrichment of these factors indicates that ferroptosis induction promotes the release of immunomodulatory signals. IL-8 and MIF, two key mediators of inflammation and tumor-immune interactions, were consistently elevated, suggesting a potential role for ferroptosis in shaping the tumor microenvironment. In contrast, downregulated proteins showed less overlap between treatments (**Figures 3D–F**), indicating a more heterogeneous suppression of secreted factors depending on the mechanism of ferroptosis induction.

**Figure 3:**
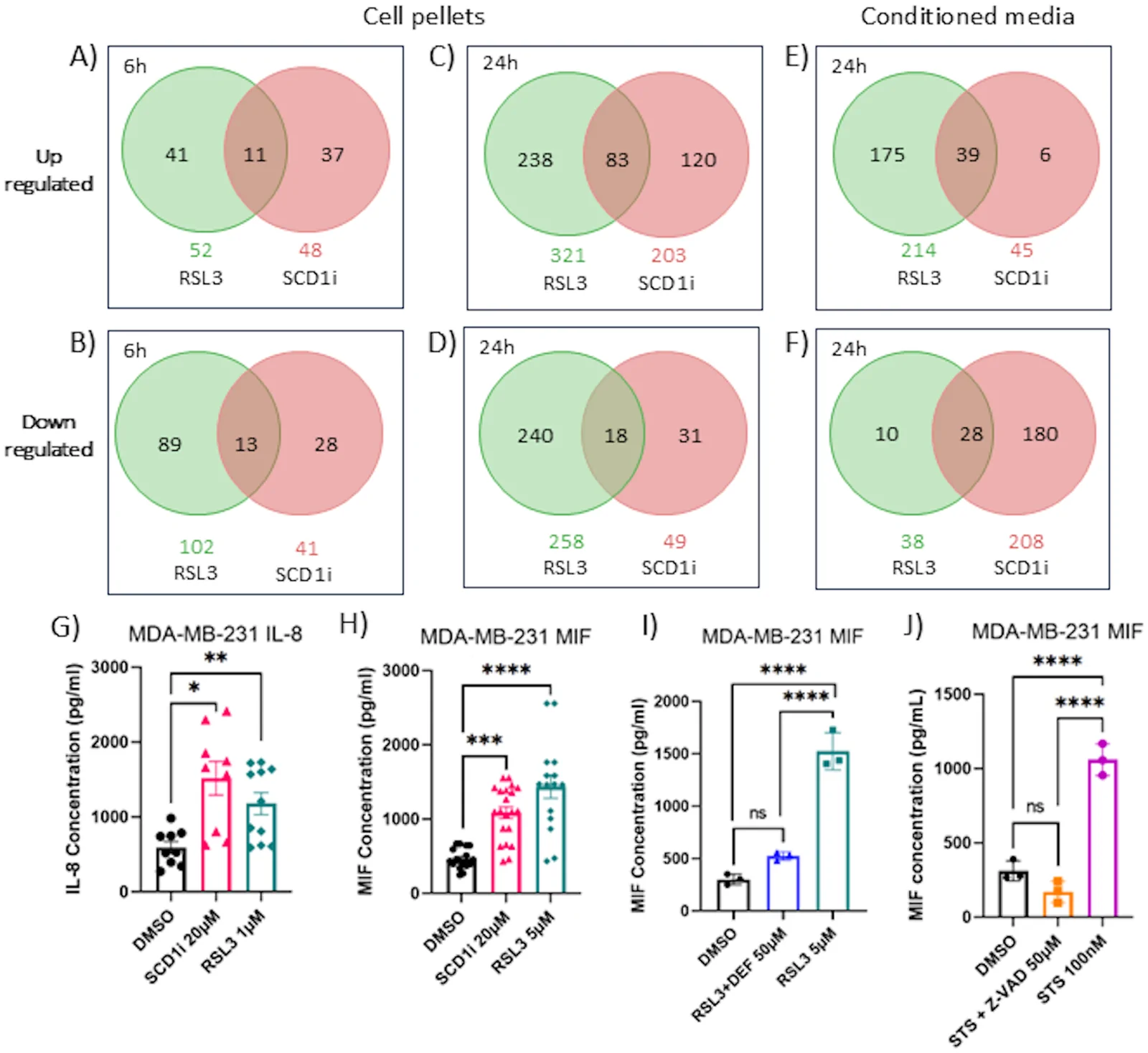
Effects of ferroptosis-inducing agents (FINs) on immunomodulation in TNBC cells. Venn diagrams show the results of proteomic analyses of **(A–D)** cell lysates and **(E–F)** conditioned media from MDA-MB-231 cells treated with FINs (RSL3 and SCD1i) for 6 h **(A, B)** or 24 h **(C–F)**. The diagrams indicate the number of proteins **(A, C, E)** upregulated and **(B, D, F)** downregulated compared to control cells treated with DMSO. Levels of IL-8 **(G)** and MIF **(H–J)** were measured in conditioned media from MDA-MB-231 cells treated with FINs **(G, H)**. MIF levels were measured in cells treated with RSL3 or in cells pretreated with ferroptosis inhibitor deferoxamine (DEF) prior to RSL3 treatment **(I)**. MIF levels were measured in cells treated with the apoptosis inducer (STS) or in cells pretreated with Z-VAD prior to STS **(J)**. Data are presented as mean ± SD. Statistical significance is indicated as *P < 0.05; **P < 0.01; ***P < 0.001; ****P < 0.0001. Each dot represents *a biological replicate*. *MIF, macrophage migration inhibitory factor; DMSO, dimethylsulfoxide; RSL3, RAS-selective lethal 3; SCD1i, stearoyl-CoA desaturase 1 inhibitor; DEF (DFO), deferoxamine; STS, staurosporine; Z-VAD*, is an irreversible, broad-spectrum pan-caspase inhibitor.

**Table 2:** Ferroptosis inducers promote the release of proteins involved in immune cell regulation.

Protein	SCDi 6h	RSL3 6h	SCD1i 24h	RSL3 24h
LAT2	14,4	1,1	1,9	0,4
HSPB11	1,4	1,4	2,2	3,2
PXDN	1,1	2,6	0,7	0,3
LAIR1	0,9	1,1	1,5	0,9
IL8	Not tested (Conditioned media)		4	0,6
MIF			0,6	1,8

Proteins found by proteomic analysis showing proteins found upregulated in cell pellets and in the conditioned of MDA-MB-231 cells treated for 6h and for 24 with RSL3 and SCD1 inhibitor highlights the modulation of immune-related proteins regulated by ferroptosis inducers. Changes are presented as log2 fold changes.

Increased MIF secretion was validated across multiple breast cancer cell lines following treatment with RSL3 and SCD1 inhibitor (**Supplementary Figure 3**). Based on these observations, we focused on IL-8 and MIF for further validation. ELISA analysis confirmed a significant increase in both IL-8 and MIF levels in the conditioned media of MDA-MB-231 cells treated for 24 h with SCD1i or RSL3(**Figures 3G, H**). Importantly, pre-treatment with the iron chelator deferoxamine markedly reduced this induction (**Figure 3I**), supporting a ferroptosis-dependent mechanism. To determine whether this effect was specific to ferroptosis, cells were treated with the apoptosis inducer staurosporine (STS). STS treatment also increased MIF secretion, and this effect was abolished by the pan-caspase inhibitor Z-VAD (**Figure 3J**), indicating that MIF release can occur downstream of distinct cell death pathways. Together, these results demonstrate that ferroptosis induction in TNBC cells not only drives extensive remodeling of the secretome but also promotes the release of key inflammatory mediators, including IL-8 and MIF.

### 3.4 Conditioned media from ferroptotic TNBC cells modulates T cell composition and activation

To evaluate the functional impact of the ferroptosis-associated secretome on immune cells, we first confirmed that MIF secretion was also increased in the murine TNBC cell line E0771 following 24 h treatment with RSL3 or SCD1i (**Figure 4A**). Splenocytes were then isolated from C57BL/6 mice and cultured overnight in conditioned media derived from treated E0771 cells, followed by flow cytometry analysis (**Figure 4B**). Immune cell profiling was performed using the gating strategy shown in **Supplementary Figure 5**. No significant differences were observed in the total number of viable cells or in the proportions of leukocytes, monocytes, macrophages, neutrophils, dendritic cells, B cells, or natural killer cells (**Supplementary Figures 6A–H**). In contrast, exposure to conditioned media from SCD1i-treated cells resulted in a significant increase in the proportion of CD3+ T lymphocytes among the CD45+ cells when compared to splenocytes cultured in control conditioned media (**Figure 4C**). Further characterization revealed that this increase was accompanied by a higher proportion of effector T cells, including both CD4^+^ helper and CD8^+^ cytotoxic subsets (**Figures 4D, E**), suggesting enhanced T cell activation or differentiation. We next assessed PD-1 expression across T cell populations. Notably, splenocytes cultured in SCD1i-conditioned media displayed a significant reduction in the proportion of PD-1 expressing cells among total CD4^+^ T cells (**Figure 4F**), effector (CD4+ CD44+ CD62L-) T cells (**Figure 5G**) and CD8^+^ effector T cells (**Figures 4F–I**), indicating a less exhausted or more activated phenotype. Reduced PD-1 expression following exposure to conditioned media from SCD1i-treated cells was also observed in myeloid cell populations, with significant decreases in PD-1^+^ macrophages, monocytes, neutrophils, and dendritic cells (**Supplementary Figures 6I–L**). Although a similar trend was observed for B cells and natural killer cells, the reduction in PD-1^+^ cells did not reach statistical significance (**Supplementary Figures 6M, N**). Collectively, these results suggest that conditioned media from ferroptotic TNBC cells, particularly following SCD1 inhibition, promotes T-cell enrichment and activation while reducing PD-1 expression across both lymphoid and myeloid immune cell populations, consistent with an immunostimulatory effect *in vitro*.

**Figure 4:**
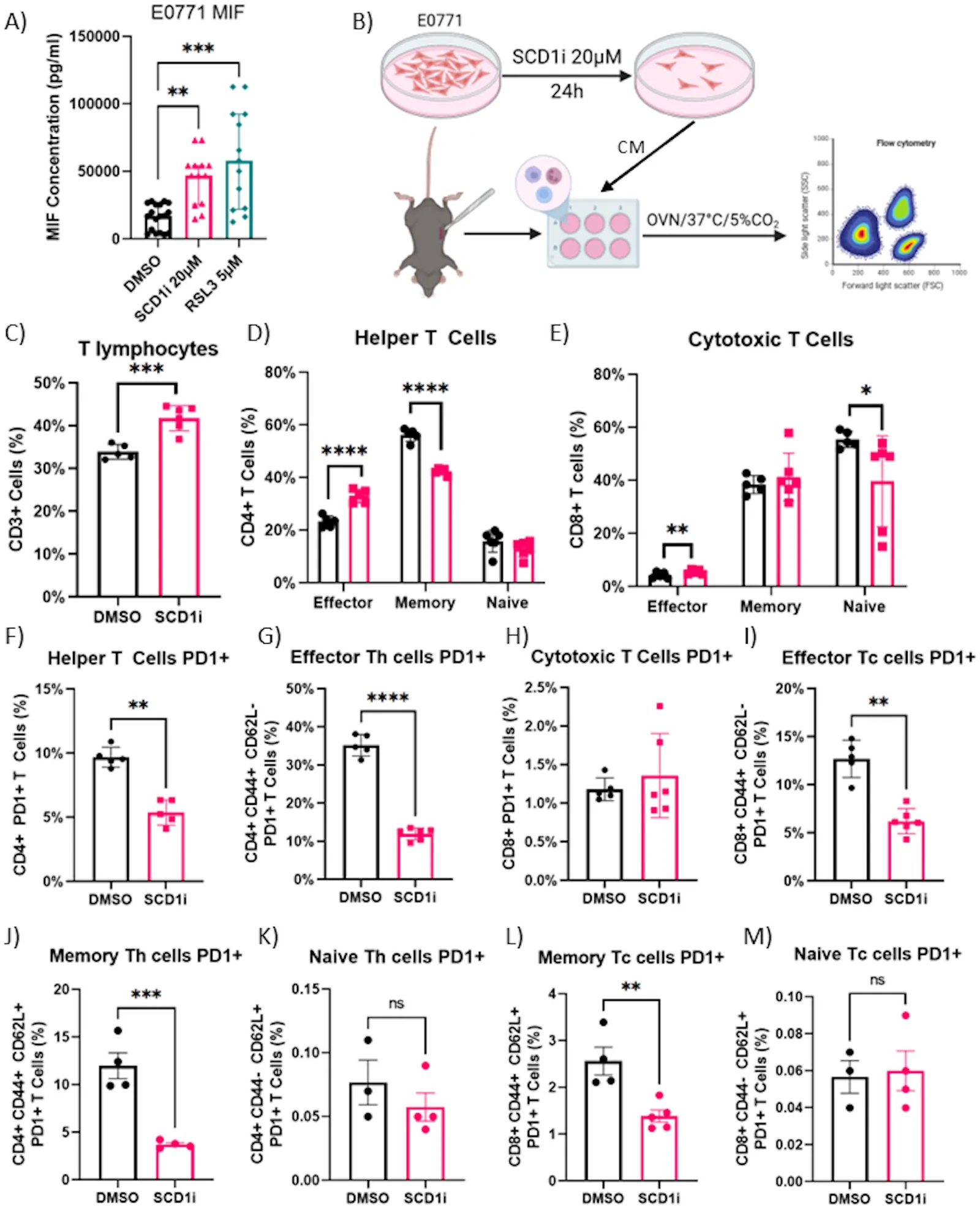
SCD1i reduces PD-1 expression on the surface of T cells in vitro. **(A)** Measurement of MIF levels in conditioned medium from C57/Black 6 TNBC cells treated with FINs for 24h. Each dot represents *a biological replicate* **(B)** Experimental model of splenocytes cultured in CM from E0771 cells treated with SCD1i, then analyzed by flow cytometry. Five spleens were used in the DMSO group (n = 5) and 6 spleens in the SCD1i group (n = 6). **(C)** Flow cytometry analysis showing the proportion of CD3+ T lymphocytes among CD45+ cells. **(D, E)** the proportion of effector, memory, and naive T lymphocytes among helper (CD4+) **(D)** and cytotoxic (CD8+) **(E)** T lymphocytes. **(F)** Proportion of PD-1+ helper (CD4+) cells. **(G)** Proportion of PD-1+ effector Th (CD4+ CD44+ CD62L-) cells. **(H)** Proportion of PD-1+ cytotoxic T cells (CD8+). **(I)** Proportion of PD-1+ effector (CD8+CD44+CD62L-) cytotoxic Tc lymphocytes. **(J–M)** Proportion of PD-1+ memory Th cells (CD4+ CD44+ CD62L+) **(J)**, PD-1+ naïve Th cells CD4+ CD44- CD62L+) **(K)**, PD-1+ memory Tc cells (CD8+CD44+CD62L+) **(L)** and PD-1+ naïve Tc cells (CD8+CD44-CD62L+) **(M)**. Results show mean ± SD. Statistical significance was determined by one-way ANOVA followed by the appropriate *post hoc* multiple-comparison test. *P<0.05; **P<0.01; ***P<0.001; ****P<0.0001 *MIF, Macrophage migration inhibitory factor; SCD1i, Stearoyl-CoA Desaturase-1 inhibitor; CM, Conditioned Medium.*.

**Figure 5:**
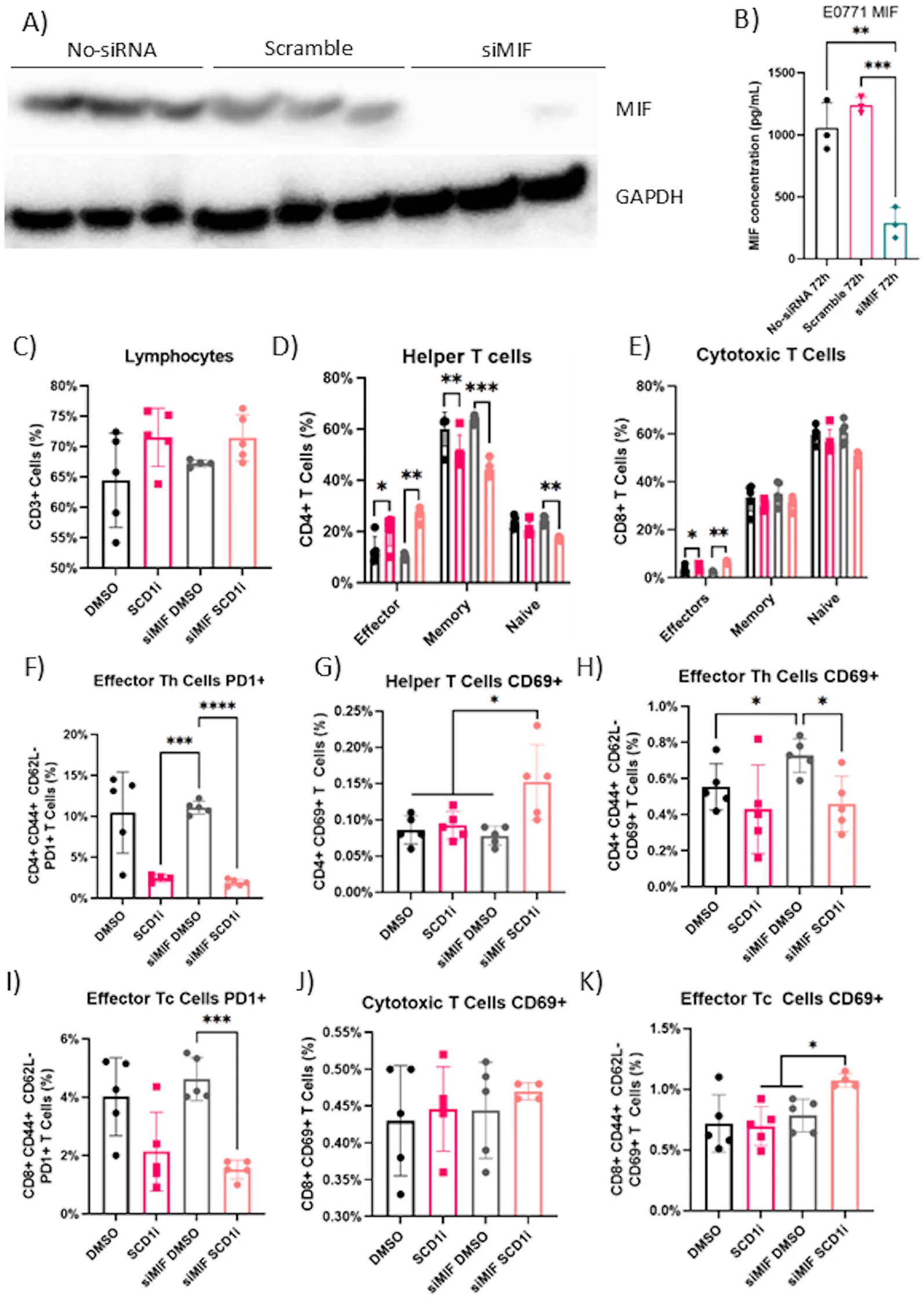
MIF silencing selectively enhances SCD1 inhibition-induced T-cell activation. **(A)** E0771 cells were transfected with MIF-specific siRNA, and MIF knockdown was confirmed by Western blot analysis of cell lysates (n = 3). **(B)** Reduced MIF secretion into the conditioned medium was confirmed by ELISA (n = 3). **(C)** Flow cytometric quantification of the proportions of naïve, effector, and memory CD4^+^ helper T cells in splenocytes incubated with conditioned media collected from E0771 cells transfected with control or MIF-specific siRNA and treated for 24 h with DMSO, the SCD1 inhibitor (SCD1i, 20 μM), MIF siRNA, or the combination of MIF siRNA and SCD1i. **(D)** Flow cytometric quantification of the proportions of naïve, effector, and memory CD8^+^ cytotoxic T cells under the same experimental conditions. **(E)** Quantification of PD-1^+^ effector CD4^+^ helper T cells. **(F)** Quantification of PD-1^+^ effector CD8^+^ cytotoxic T (Tc) cells. **(G)** Quantification of CD69^+^ CD4^+^ helper T cells. **(H)** Quantification of CD69^+^ effector CD8^+^ cytotoxic T (Tc) cells (n = 5 spleens). Data are presented as mean ± SD. Statistical significance was determined by one-way ANOVA followed by the appropriate *post hoc* multiple-comparison test. *P < 0.05; **P < 0.01; ***P < 0.001.

### 3.5 MIF silencing selectively enhances SCD1 inhibition-induced T-cell activation

Since treatment of E0771 cells with the SCD1 inhibitor (SCD1i) increased MIF production and enhanced splenocyte activation, we investigated whether simultaneous inhibition of SCD1 and MIF could further modulate the immune response. E0771 cells were transfected with MIF-specific siRNA, and efficient MIF knockdown was confirmed by Western blot analysis of cell lysates (**Figure 5A**). Reduced MIF secretion into the conditioned media was confirmed by ELISA (**Figure 5B**). Conditioned media from control- and MIF siRNA-transfected E0771 cells treated with vehicle (DMSO) or SCD1i were then collected and used to evaluate their effects on splenocytes. Flow cytometric analysis showed that MIF knockdown alone had no significant effect on splenocyte phenotype, including the proportions of naïve, effector, and memory CD4^+^ (**Figure 5C**) and CD8^+^ (**Figure 5D**) T-cell subsets. Likewise, MIF silencing did not alter PD-1 expression on either CD4^+^ or CD8^+^ T cells. In contrast, conditioned media from SCD1i-treated cells increased the proportion of effector CD4^+^ and CD8^+^ T cells (**Figures 5C, D**) and, consistent with our findings using conditioned media from parental E0771 cells (**Figure 4**), significantly reduced the proportion of PD-1^+^ CD4^+^ and PD-1^+^ CD8^+^ T cells (**Figures 5E, F**). MIF knockdown did not further modify these effects of SCD1 inhibition, suggesting that MIF is not a major mediator of the effects of SCD1 inhibition on T-cell phenotype or PD-1 expression under these experimental conditions. In contrast, combined MIF knockdown and SCD1 inhibition significantly increased the proportion of CD69^+^ CD4^+^ T cells compared with control, SCD1 inhibition, and MIF siRNA alone (**Figure 5G**). A similar trend was observed for CD69^+^ CD8^+^ T cells; although this increase did not reach statistical significance compared with the control group, it was significant compared with both SCD1 inhibition and MIF siRNA alone (**Figure 5H**). Together, these findings suggest that, while MIF does not substantially influence the effects of SCD1 inhibition on T-cell differentiation or PD-1 expression, it may partially restrain SCD1 inhibition-induced T-cell activation, as reflected by the increased frequency of CD69^+^ T cells following combined SCD1 inhibition and MIF silencing.

### 3.6 Inhibition of SCD1 *in vivo* enhances tumor infiltration by PD-1 positive cytotoxic T cells

We investigated the effect of SCD1 inhibition on tumor growth using the syngeneic TNBC model in which E0771 cells were implanted into C57BL/6 mice. Treatment with the SCD1 inhibitor (SCD1i), which promotes lipid peroxidation, resulted in a significant reduction in tumor growth compared to vehicle-treated mice (**Figures 6A, B**). SCD1 inhibition did not result in any significant body weight loss (**Figure 6C**), indicating that the treatment was well tolerated under these experimental conditions. Tumors were subsequently harvested and analyzed by FACS using the gating strategy shown in **Supplementary Figure 7**. There was no significant difference in the viability of tumor-infiltrating cells, indicating that SCD1 inhibition did not compromise the overall viability of tumor-infiltrating immune cells (**Figure 6D**). We observed a significant increase in the proportion of T lymphocytes (CD3+) among CD45+ cells in SCD1i-treated tumors (**Figure 6E**), with a specific increase in cytotoxic (CD8+) T lymphocytes (CTLs) (**Figure 6F**). Moreover, the fraction of CTLs expressing the immune checkpoint markers PD-1 and LAG-3 was significantly higher across effector, memory, and naïve CD8+ T-cell subsets in tumors treated with SCD1i (**Figures 6G–N**). The effect of SCD1 inhibition *in vivo* was assessed by immunohistochemical staining for 4-hydroxynonenal (4-HNE). Representative images and quantification showed markedly increased 4-HNE accumulation in the cytoplasm of tumor cells from SCD1 inhibitor-treated mice compared with control mice (**Figures 6O, P**), supporting increased lipid peroxidation *in vivo* and corroborating the *in vitro* findings shown in **Figure 2**.

**Figure 6:**
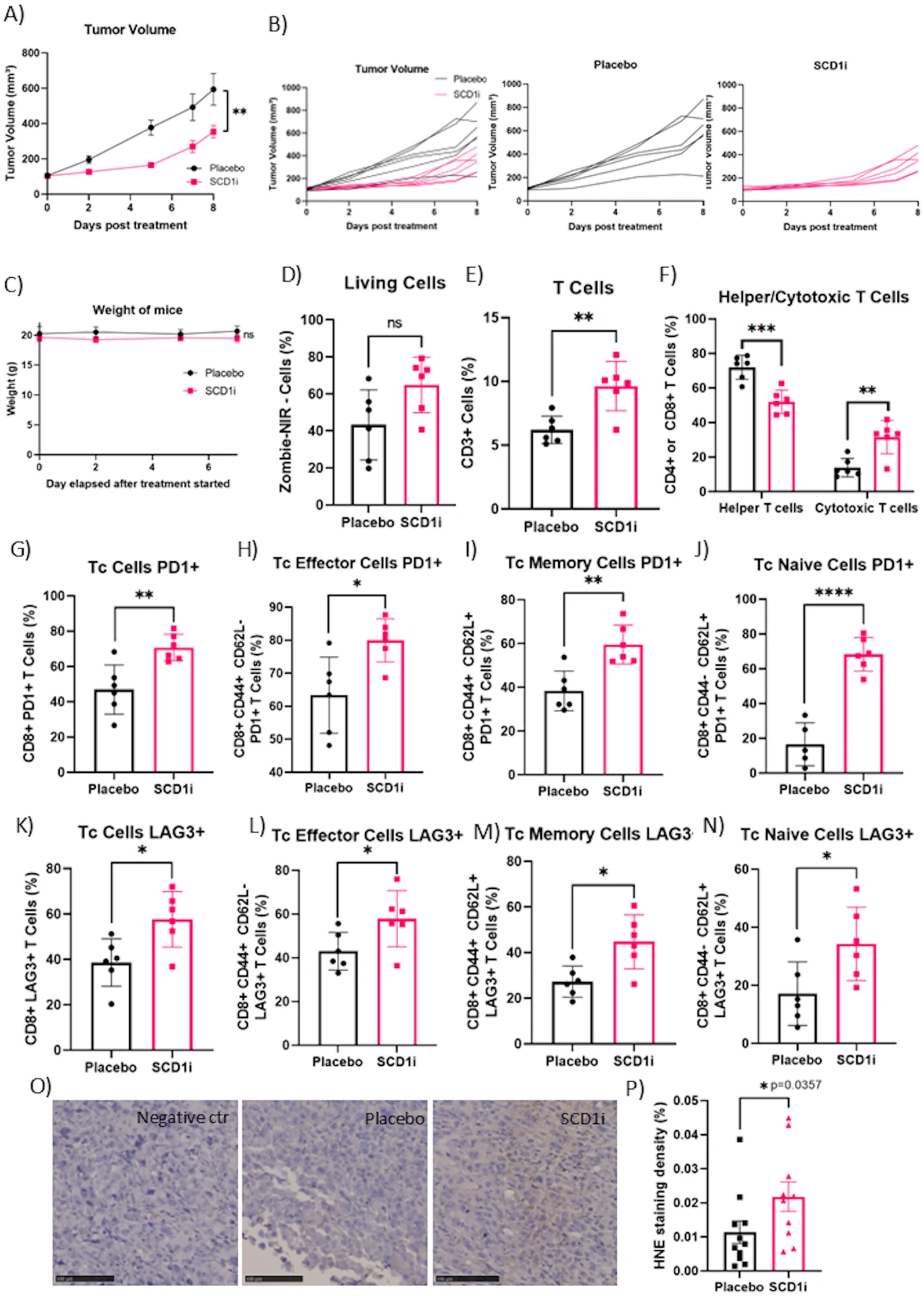
SCD1 inhibition increases PD-1 and LAG-3 expression on the surface of cytotoxic T cells *in vivo*. **(A)** Tumor growth curves of mouse E0771 tumors injected *s.c.* in C57/Black6 mice (n = 6 per group) and treated for 7 days with SCD1i or vehicle control (placebo). **(B)** Individual tumor growth curves (spaghetti plots) showing the tumor evolution of individual mice in the two pooled groups (left), the DMSO-treated group (middle), and the SCD1i-treated group (right). **(C)** Mouse body weight following treatment with vehicle or SCD1i. **(D)** the number of living cells within the tumor, **(E)** the proportion of T lymphocytes (CD3+) among leukocytes (CD45+), **(F)** the proportions of CD4+ and CD8+ T lymphocytes among T lymphocytes (CD3+), **(G–J)** the proportion of PD-1+ cytotoxic T lymphocytes (CD8+) **(G)**, PD-1+ cytotoxic effector (CD8+CD44+CD62L-) **(H)**, PD-1+ memory (CD8+CD44+CD62L+) **(I)**, and PD-1+ naïve (CD8+CD44-CD62L+) T cells **(J)**. K-N) the proportion of LAG-3+ cytotoxic T lymphocytes **(K)**, LAG-3+ cytotoxic effector **(L)**, LAG-3+ memory **(M)**, and LAG-3+ naive T cells **(N)**. **(O)** Representative 4-HNE staining in the negative control (no antibody), vehicle-treated, and SCD1 inhibitor-treated E0771 tumors. **(P)** Quantification of 4-HNE staining density in whole tumor sections from tumors obtained from mice treated with SCD1i (n = 10) or vehicle (n = 11) across two independent experiments. Staining density was normalized to the maximum measured value, which was set to 1.0 (100%). Statistical significance was determined using unpaired t test or Mann-Whitney test. The significance of tumor growth was calculated using a t-test for each day measured. Results show mean ± SD, *P<0.05; **P<0.01; ***P<0.001; ****P<0.0001. For flow cytometry analyses, n = 6 mice per group; the two tumors from each mouse were pooled, resulting in one biological replicate per mouse. SCD1i, Stearoyl-CoA Desaturase-1 inhibitor.

Overall, SCD1 inhibition reduced tumor growth and increased cytotoxic T-cell infiltration, accompanied by increased expression of PD-1 and LAG-3 on these cells and increased PD-1 expression across several additional immune populations. These findings provide a rationale for combining SCD1 inhibition with immune checkpoint blockade in this TNBC model.

#### 3.6.1 SCD1 inhibition potentiates anti-PD-1 efficacy in the E0771 syngeneic model

To evaluate whether ferroptosis induction enhances the response to immune checkpoint blockade, we assessed the therapeutic efficacy of SCD1 inhibition in combination with anti-PD-1 in the E0771 syngeneic breast cancer model. C57BL/6 tumor-bearing mice (tumor volume 70–100 mm³) were randomized into four groups and treated with vehicle control (DMSO + IgG), SCD1 inhibitor (SCD1i; 40 mg/kg by oral gavage), anti-PD-1 antibody (10 mg/kg by intraperitoneal injection; 0.2 mg per injection per mouse, twice weekly), or the combination of SCD1i and anti-PD-1 (**Figure 7**). Analysis of tumor growth curves revealed that anti-PD-1 monotherapy did not significantly reduced tumor progression, whereas SCD1 inhibition significantly reduces tumor growth compared to placebo over the course of the experiment (**Figures 7A–C**). In contrast, the combination treatment resulted in a more pronounced attenuation of tumor growth than either single agent treatment, which became evident at later time points (**Figures 7A–D**). To integrate tumor growth over time, area under the curve (AUC) analysis was performed (**Figure 7E**). This analysis confirmed a significant decrease in overall tumor burden in the combination group, supporting an enhanced therapeutic effect when SCD1 inhibition is combined with immune checkpoint blockade. Consistent with increased T cell infiltration and elevated expression of checkpoint receptors following SCD1 inhibition (**Figures 6E–N**), these findings support a model in which SCD1 inhibition and associated lipid peroxidation may contribute to increased tumor responsiveness to immune checkpoint blockade.

**Figure 7:**
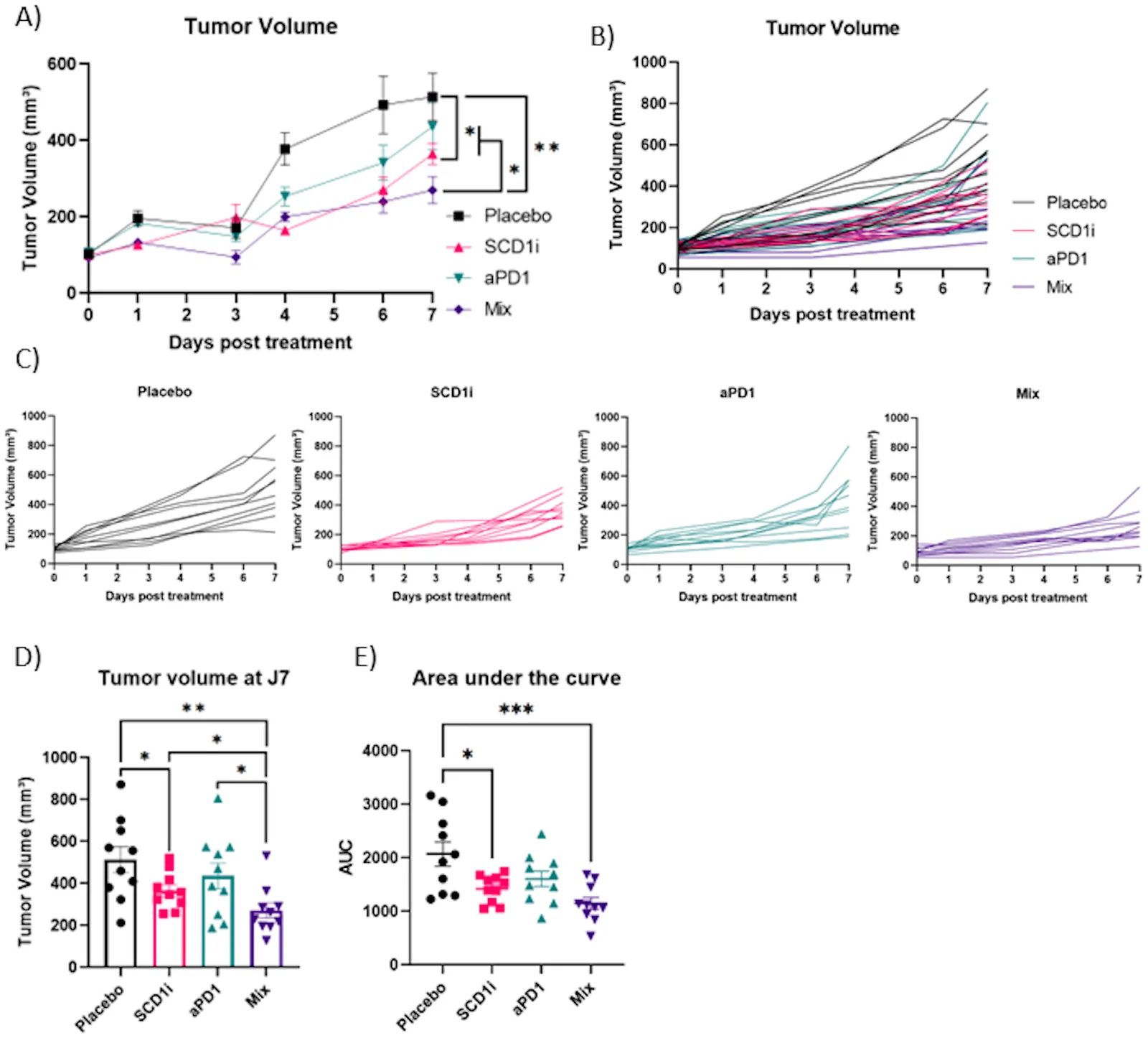
SCD1 inhibition enhances the efficacy of anti-PD-1 therapy in the E0771 syngeneic model. **(A)** Tumor growth curves of E0771 tumors bearing C57BL/6 mice were treated with vehicle control (placebo), SCD1 inhibitor (SCD1i), anti-PD-1 antibody (aPD-1), or the combination of SCD1i and anti-PD-1 (Mix). **(B)** Individual tumor growth curves (spaghetti plots) showing the tumor evolution of individual mice in the four pooled groups. **(C)** Spaghetti plots for each group are shown as indicated. **(D)** Tumor volume at day 7 post-treatment. Each dot represents an individual mouse; bars indicate mean ± SEM. **(E)** Area under the curve of tumor volume in **(A)**. Statistical significance was determined using one-way ANOVA with Tukey multiple comparisons. The AUC for each tumor was calculated using GraphPad Prism 9.0 software and plotted as the mean ± SD. (n = 10 mice) *P < 0.05, ***P < 0.001, ****P < 0.0001.

Collectively, these results indicate that SCD1 inhibition sensitizes tumors to anti-PD-1 therapy, leading to improved tumor control *in vivo*, while having limited efficacy as a single agent under these experimental conditions.

## 4 Discussion

In this study, we demonstrate that ferroptosis induction in breast cancer not only drives tumor cell death but also profoundly remodels the tumor microenvironment, with significant implications for anti-tumor immunity and therapeutic response. By combining pharmacological approaches targeting canonical ferroptosis pathways and lipid metabolism, we identify subtype-specific vulnerabilities and uncover an immunomodulatory role for the ferroptotic secretome.

Ferroptosis has emerged as a distinct, iron-dependent form of regulated cell death driven by lipid peroxidation, with increasing relevance in cancer biology and therapy (15, 16). In the present study, we show that ferroptosis induction in breast cancer not only exerts tumor cell–intrinsic cytotoxic effects but also profoundly reshapes the tumor microenvironment, particularly through modulation of the tumor secretome and immune landscape.

Consistent with prior studies, we observed that direct inhibition of GPX4 using RSL3 is a highly potent inducer of ferroptosis across breast cancer cell lines (17, 18). In contrast, targeting lipid metabolism via SCD1 inhibition resulted in more moderate effects, in line with reports highlighting the role of MUFAs in buffering lipid peroxidation and ferroptotic sensitivity (19, 20). Notably, TNBC cells were particularly sensitive to both ferroptosis induction and SCD1 inhibition, supporting previous observations that aggressive and mesenchymal-like cancer states are more vulnerable to ferroptosis (21).

Beyond cytotoxicity, our findings demonstrate that ferroptosis induces a marked remodeling of the tumor secretome. Proteomic analysis revealed a conserved set of proteins upregulated across distinct ferroptosis-inducing treatments, including several factors implicated in immune regulation. Among these, IL-8 and MIF were consistently elevated. Both cytokines are well-established mediators of inflammation and tumor–immune interactions, with IL-8 linked to neutrophil recruitment and immunosuppression, and MIF implicated in macrophage polarization and tumor progression (22, 23). Recent studies have begun to suggest that ferroptotic cells can release damage-associated molecular patterns (DAMPs) and cytokines capable of modulating immune responses (13, 24), and our data extend these findings by identifying a specific ferroptosis-associated secretory profile in TNBC models.

Functionally, splenocytes incubated with conditioned media from ferroptotic cells promoted T cell enrichment and activation *in vitro*, as evidenced by increased proportions of effector T cells and reduced PD-1 expression in both lymphoid and myeloid cells. These observations are consistent with emerging evidence that ferroptosis can exert immunogenic effects under certain contexts (25, 26). Our findings identify MIF as a component of the ferroptosis-associated secretome induced by SCD1 inhibition that partially restrains the immune-stimulatory effects of ferroptotic tumor cells. Although SCD1 inhibition increased MIF secretion, conditioned media from MIF-depleted cells further enhanced CD69 expression on both CD4^+^ and CD8^+^ T cells without further affecting PD-1 expression. These results suggest that, while SCD1 inhibition triggers the release of immunostimulatory signals capable of promoting T-cell activation, it simultaneously induces compensatory factors such as MIF that may limit the magnitude of this response. This is consistent with the well-established immunosuppressive functions of MIF in the tumor microenvironment, where it promotes immune evasion by suppressing T-cell activation and shaping myeloid cell function (27). The lack of effect of MIF knockdown alone further suggests that MIF is not a dominant regulator of basal immune activity in this model but rather modulates the immune response elicited by ferroptotic stress. Together, these findings support a model in which the ferroptosis-associated secretome contains both immunostimulatory and immunoregulatory mediators and indicate that simultaneous targeting of SCD1 and MIF may enhance the immune-activating consequences of ferroptosis induction. However, our *in vivo* data reveal a more complex scenario. While SCD1 inhibition reduced tumor growth and increased infiltration of cytotoxic T lymphocytes, it was also associated with elevated expression of PD-1 and LAG-3 on tumor-infiltrating CD8^+^ T cells, consistent with the development of an exhausted T-cell phenotype (28). This dual phenotype characterized by both activation and checkpoint upregulation is a hallmark of chronic antigen exposure and has been widely described in tumor-infiltrating T cells (29). These findings suggest that ferroptosis induction by SCD1 inhibition creates a pro-inflammatory environment that promotes T-cell recruitment and activation while simultaneously eliciting compensatory immunosuppressive mechanisms that may limit sustained antitumor immunity. This observation provided the rationale for combining SCD1 inhibition with PD-1 immune checkpoint blockade in the E0771 tumor model. An apparent difference was observed between our *in vitro* and *in vivo* immune findings. Conditioned media from SCD1 inhibitor-treated tumor cells reduced the proportion of PD-1 expressing T cells while promoting T-cell activation in the splenocyte assay. In contrast, *in vivo* SCD1 inhibition increased T-cell infiltration into the tumor microenvironment and was accompanied by increased expression of the inhibitory receptors PD-1 and LAG-3 on CD4+ and CD8+ T cells. Although increased expression of these receptors may reflect a compensatory immunoregulatory response following enhanced T-cell activation, it does not by itself establish T-cell exhaustion. PD-1 and LAG-3 is dynamically regulated and can occur during early T-cell activation, while sustained antigen exposure and chronic inflammatory signaling within the tumor microenvironment may promote persistent checkpoint receptor expression as an adaptive negative feedback response (30–32). Thus, our findings are more appropriately interpreted as evidence of increased checkpoint signaling rather than definitive T-cell exhaustion. Interestingly, the increase in PD-1 expression following SCD1 inhibition was not restricted to T cells but extended to multiple immune populations within the tumor microenvironment, including macrophages, monocytes, dendritic cells, and NK cells. These findings suggest that SCD1 inhibition induces broader remodeling of immune checkpoint signaling involving both lymphoid and myeloid/innate immune populations. Such checkpoint upregulation may represent a compensatory immunoregulatory response to the altered tumor microenvironment following SCD1 inhibition and may limit the full antitumor activity of SCD1 inhibition alone. Consistent with this interpretation, combining SCD1 inhibition with anti-PD-1 therapy resulted in greater tumor growth inhibition than either treatment alone, supporting the therapeutic potential of targeting SCD1 together with immune checkpoint blockade. Although short-term treatment with either SCD1 inhibition or anti-PD-1 monotherapy produced only modest antitumor effects, their combination enhanced tumor control in the E0771 preclinical model. While *in vivo* rescue experiments would be essential to establish whether the immunomodulatory effects of SCD1 inhibition are causally linked to ferroptosis and the observed immune remodeling, our findings are consistent with recent reports demonstrating that ferroptosis induction can sensitize tumors to immune checkpoint blockade (26). Mechanistically, ferroptosis may enhance tumor immunogenicity and immune infiltration while simultaneously increasing checkpoint expression, thereby creating a therapeutic window in which checkpoint blockade can maximize the antitumor immune response.

From a translational perspective, targeting lipid metabolism enzymes such as SCD1 represents a promising strategy to modulate ferroptosis sensitivity in tumors. SCD1 inhibitors are currently under investigation in preclinical and early clinical settings (33–35), and our data suggest that their combination with immunotherapy may be particularly relevant in TNBC, a subtype with limited targeted treatment options. These findings are consistent with the report by Wang et al. (36), demonstrating that SCD1 is regulated by Wnt/β-catenin signaling, and that inhibition of this pathway improves the efficacy of anti-PD-1 immunotherapy in preclinical skin cancer models (36). However, the dual role of factors such as IL-8 and MIF capable of promoting both immune activation and tumor progression highlights the need for a deeper mechanistic understanding of ferroptosis-induced signaling in the tumor microenvironment.

## 5 Conclusion

In conclusion, our study supports a model in which ferroptosis acts as a bridge between tumor cell death and immune modulation. By coupling lipid peroxidation–driven cytotoxicity with the release of immunologically active factors, ferroptosis induction reshapes the tumor microenvironment in a manner that can be therapeutically exploited. Future work will be required to dissect the specific mediators of this crosstalk and to optimize combinatorial strategies integrating ferroptosis induction with immune checkpoint blockade in breast cancer.

## Data Availability

Proteomic analysis of TNBC cells treated with FINs was deposited in PRIDE via the ProteomeXchange Consortium under the identifier PXD082708. Further information and requests for resources and reagents should be directed to and will be fulfilled by the corresponding author, Dr. Nor Eddine Sounni.
