# Supplementary material for: SCD1 inhibition promotes lipid peroxidation and enhances anti-PD-1 response in triple-negative breast cancer
Source: Front Immunol. 2026 Oct 12;17:1843935. doi: 10.3389/fimmu.2026.1843935 (PMC13696522; doi:10.3389/fimmu.2026.1843935)
Supplement: Supplementary file 1 [file SupplementaryFile1.pdf]

Supplementary Material

1 Supplementary Data

Supplementary Material should be uploaded separately on submission. Please include any supplementary data, figures and/or tables.

Supplementary material is not typeset so please ensure that all information is clearly presented, the appropriate caption is included in the file and not in the manuscript, and that the style conforms to the rest of the article.

2 Supplementary Figures and Tables

For more information on Supplementary Material and for details on the different file types accepted, please see here.

2.1 Supplementary Figures

SCD1 inhibition promotes lipid peroxidation and enhances anti-PD-1 response in triple-negative breast cancer

Quentin Herrera Garfia[1#], Aurélien Azam[1#], Eric Bonneil[2], Laetitia Montero-Ruiz[1,3], Mohsen Momeini [1], Mohmmad Farran [1], Robin Jehay[1] and Nor Eddine Sounni[1*]

#Contributed equally

[1]*Cancer Metabolism and Tumor Microenvironment Lab, Interdisciplinary Group for Applied Genoproteomics, University of Liège, Liège, Belgium*

[2] *Institute for Research in Immunology and Cancer (IRIC), University of Montréal, Montréal, Canada*

[3]*Laboratory of Tumor and Development Biology, Interdisciplinary Group for Applied Genoproteomics, University of Liège, Liège, Belgium*

*** Correspondence:**
Nor Eddine Sounni
nesounni@uliege.be

Keywords: TNBC, Immunotherapy, SCD1, MIF, anti-PD-1, Ferroptosis, Ferroptosis inducers. (Min.5-Max. 8)

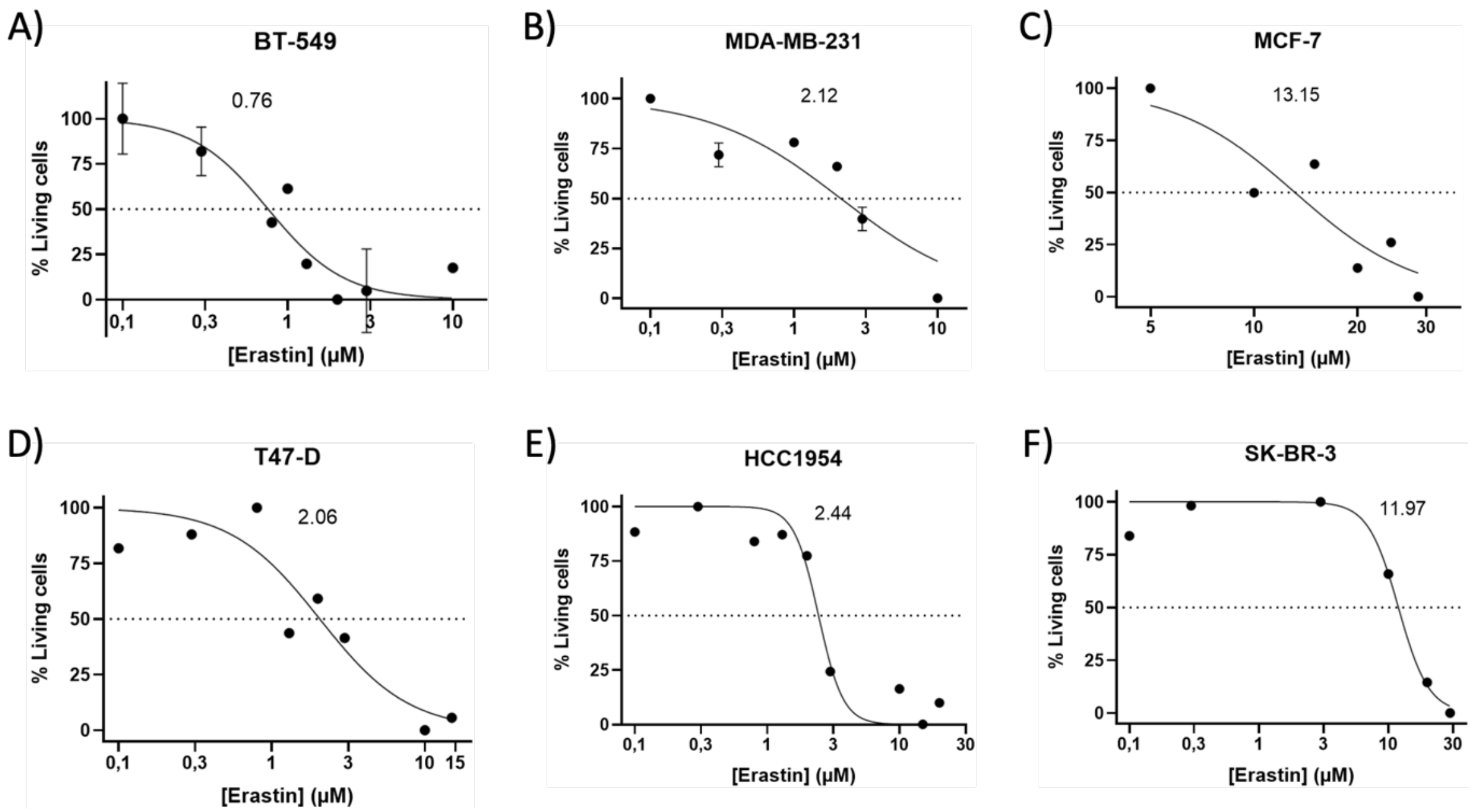

Supplementary Figure 1. Sensitivity of breast cancer cells to erastin-induced ferroptosis. Breast cancer cell lines (indicated) were treated with increasing concentrations of erastin (A–F). Cell proliferation was monitored by IncuCyte live-cell imaging, and growth inhibition was determined based on the number of viable cells (n = 3).

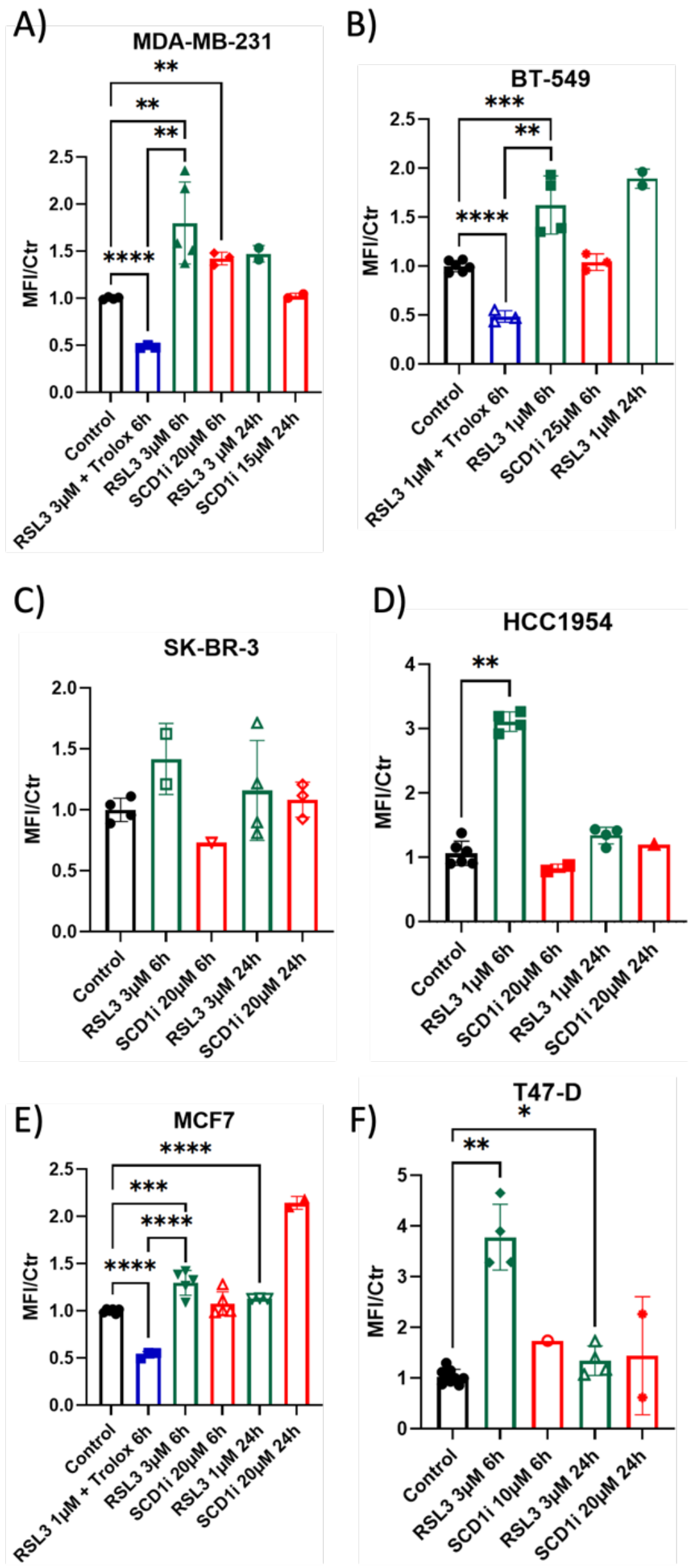

Supplementary Figure 2. SCD1 inhibition increases lipid peroxidation in breast cancer cells. Lipid peroxidation was assessed by flow cytometry using the fluorescent lipid peroxide probe BODIPY™ 581/591 C11. A, B) Quantification of lipid peroxidation, expressed as mean fluorescence intensity (MFI) relative to control (Ctr), in MDA-MB-231 (A) and BT-549 (B) cells. C–F) Lipid peroxidation in SK-BR-3 (C), HCC1954 (D), MCF7 (E), and T47D (F) cells treated with vehicle, RSL3, or the SCD1 inhibitor (SCD1i). Where indicated, cells were pretreated with Trolox before RSL3 treatment. The data represent the mean fluorescence intensity (MFI) of C11 fluorescence**,** and the replicates shown are biological replicates**.** Data are presented as mean ± SD. Statistical significance is indicated as **P < 0.01; ***P < 0.001; ****P < 0.0001.

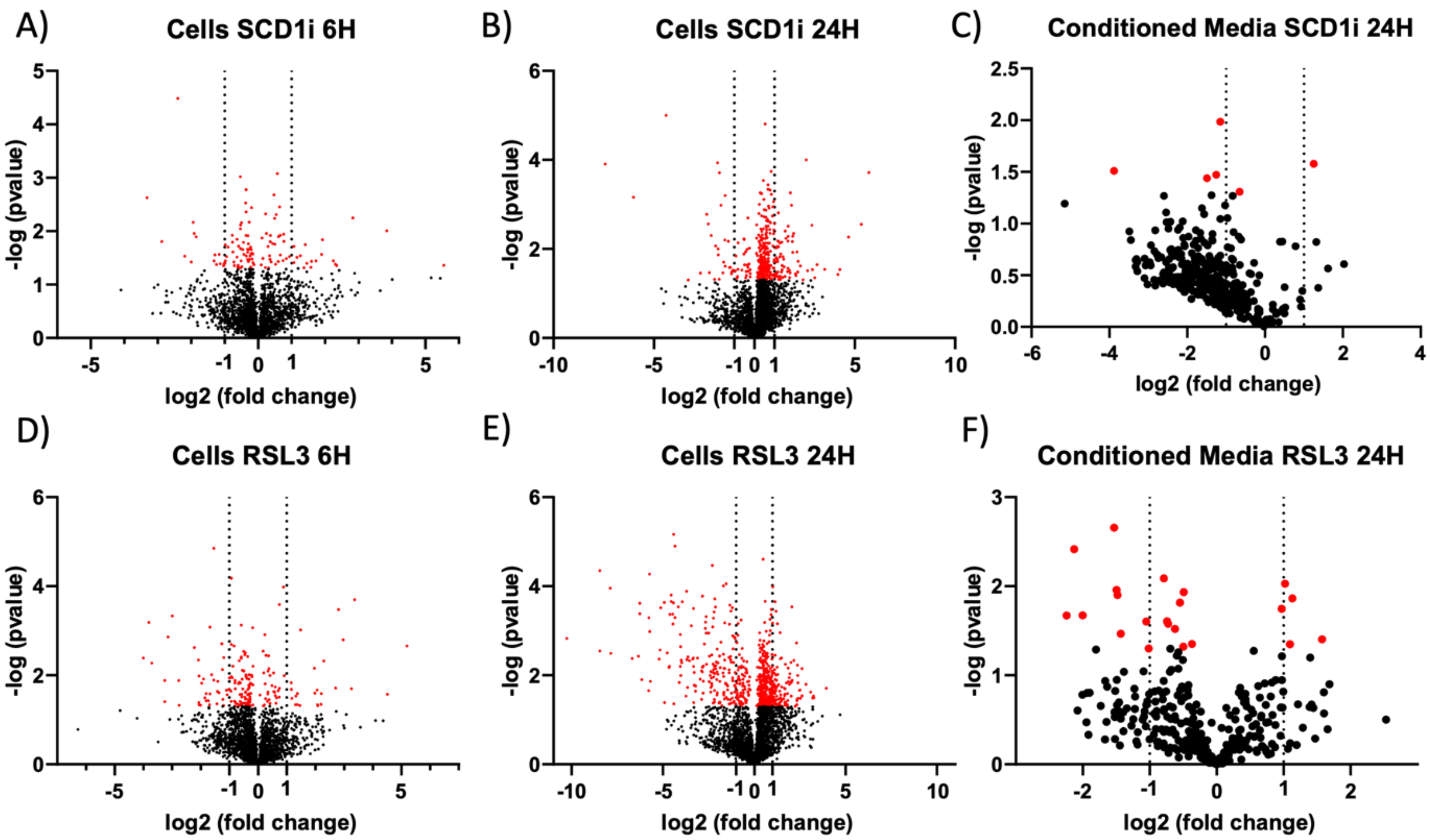

Supplementary Figure 3. **Proteomic analysis of MDA-MB-231 cells treated with ferroptosis-inducing agents (FINs).**

Volcano plots showing differentially expressed proteins in cell lysates **(A, B, D, E)** and conditioned media (C, F) from MDA-MB-231 cells treated with the ferroptosis-inducing agents (FINs) SCD1 inhibitor (SCD1i) (A–C) or RSL3 (D–F) for 6 h (A, C, D, F) or 24 h (B, E), compared with vehicle-treated (DMSO) controls. Proteins showing statistically significant differential expression are highlighted in red and fall outside the vertical threshold lines. Changes are presented as log2 fold change.

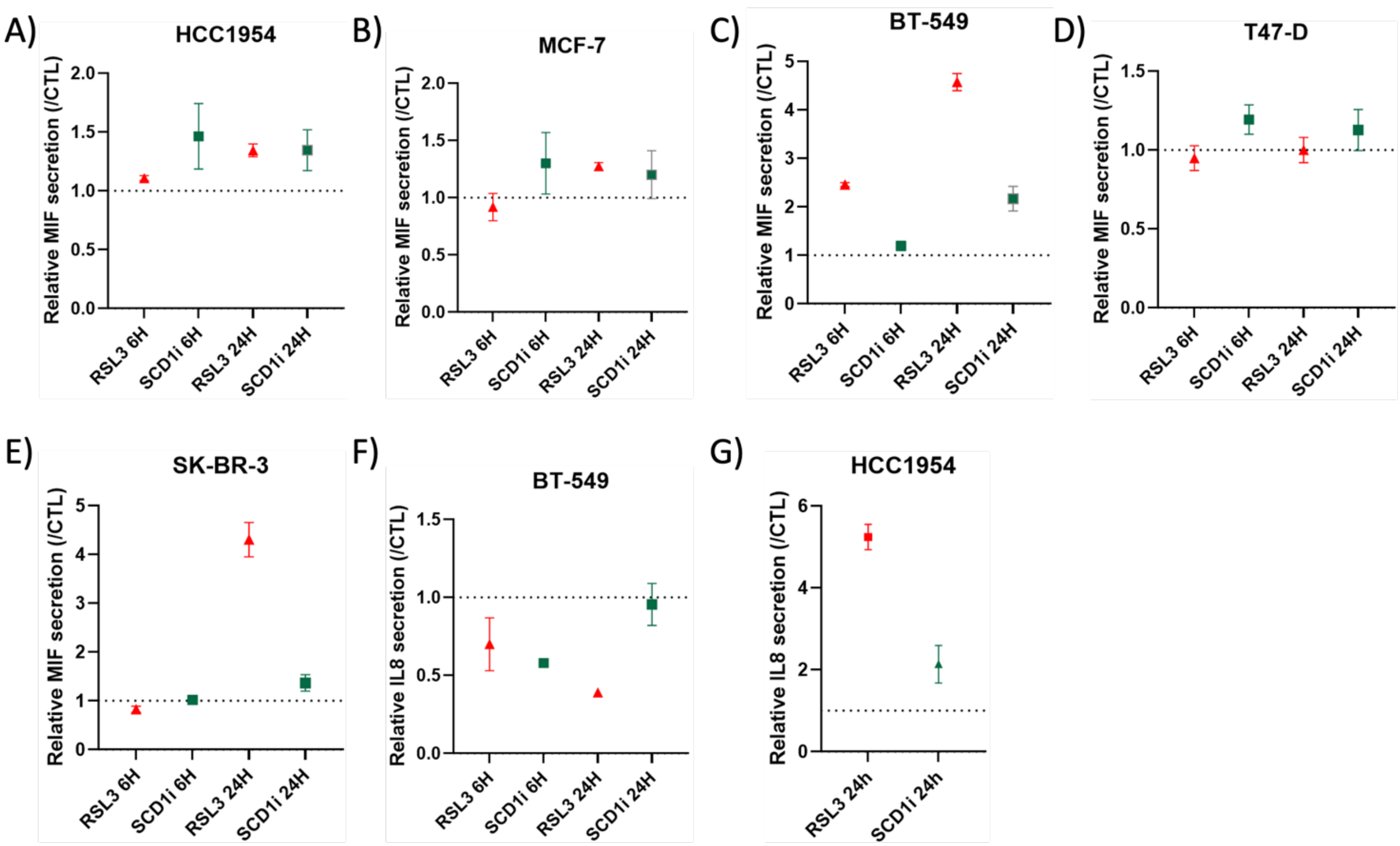

Supplementary Figure 4. Ferroptosis inducers promote MIF secretion in the conditioned media of multiple breast cancer cell lines.
A–E) Quantification of MIF secretion by ELISA in the conditioned media of the indicated breast cancer cell lines treated with the ferroptosis inducers RSL3 or SCD1 inhibitor (SCD1i) for 6 or 24 h. F–G**)** Quantification of IL-8 levels by ELISA in the conditioned media of breast cancer cell lines treated with the same ferroptosis inducers as in A–E. Data are presented as mean ± SD (n = 3 biological replicates).

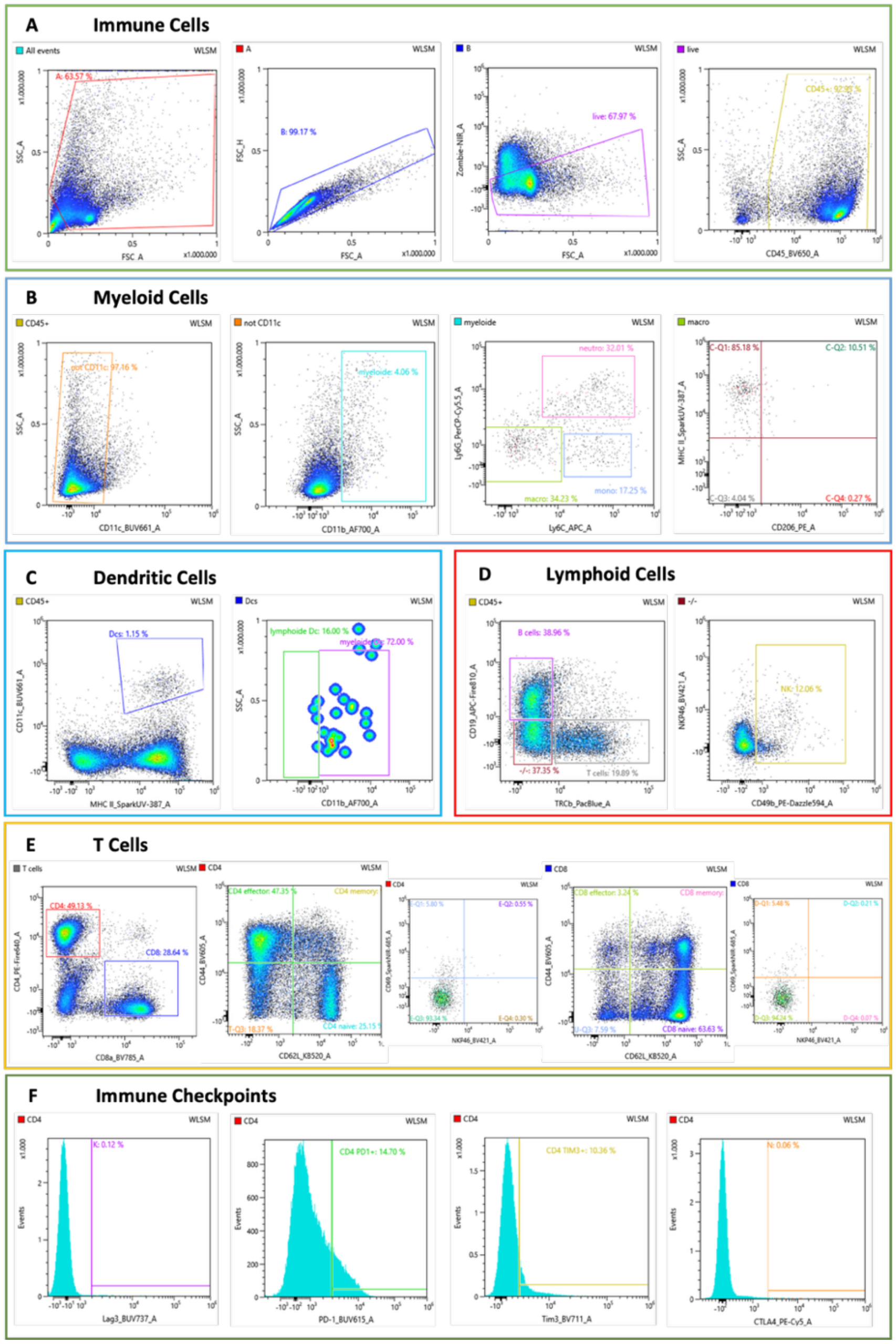

Supplementary Figure 5. **Flow Cytometry Gating of splenocytes.** A) Cells were first gated for morphology (FSC-A/SSC-A) and single cells (FSC-A/FSC-H). Cells were then labeled with Zombie-Nir for cell viability. The cells were then labeled with a CD45 marker for leukocytes. B) Myeloid Cells were gated first on CD11c-, then CD11b+. Macrophages were gated with Ly6G low and Ly6C low, monocytes were Ly6G low and Ly6C high, and neutrophiles were gated with Ly6G high and Ly6C high. Macrophages were gated with CD206 and MHCII to differentiate M1 like and M2 like. C) Dendritic cells were gated in CD11c+ and MHCII+ cells. Lymphoid and myeloid DC were differentiated with CD11b. D) Lymphoid cells were gated from CD45+ cells. B cells were CD19+, and T cells were TCRb+. NK cells were CD19-/TCRb- and CD49b+. E) T cells were differentiated into Helper (CD4+) and Cytotoxic CD8+) T Cells. These two populations were differentiated into CD4+ or CD8+ effector (CD44+/CD62L-), memory (CD44+/CD62L+), and naïve (CD44-/CD62L+) T lymphocytes. F) Cells were also labeled with markers for CD69 PD1, LAG-3, Tim-3, CTLA-4 and BTLA.

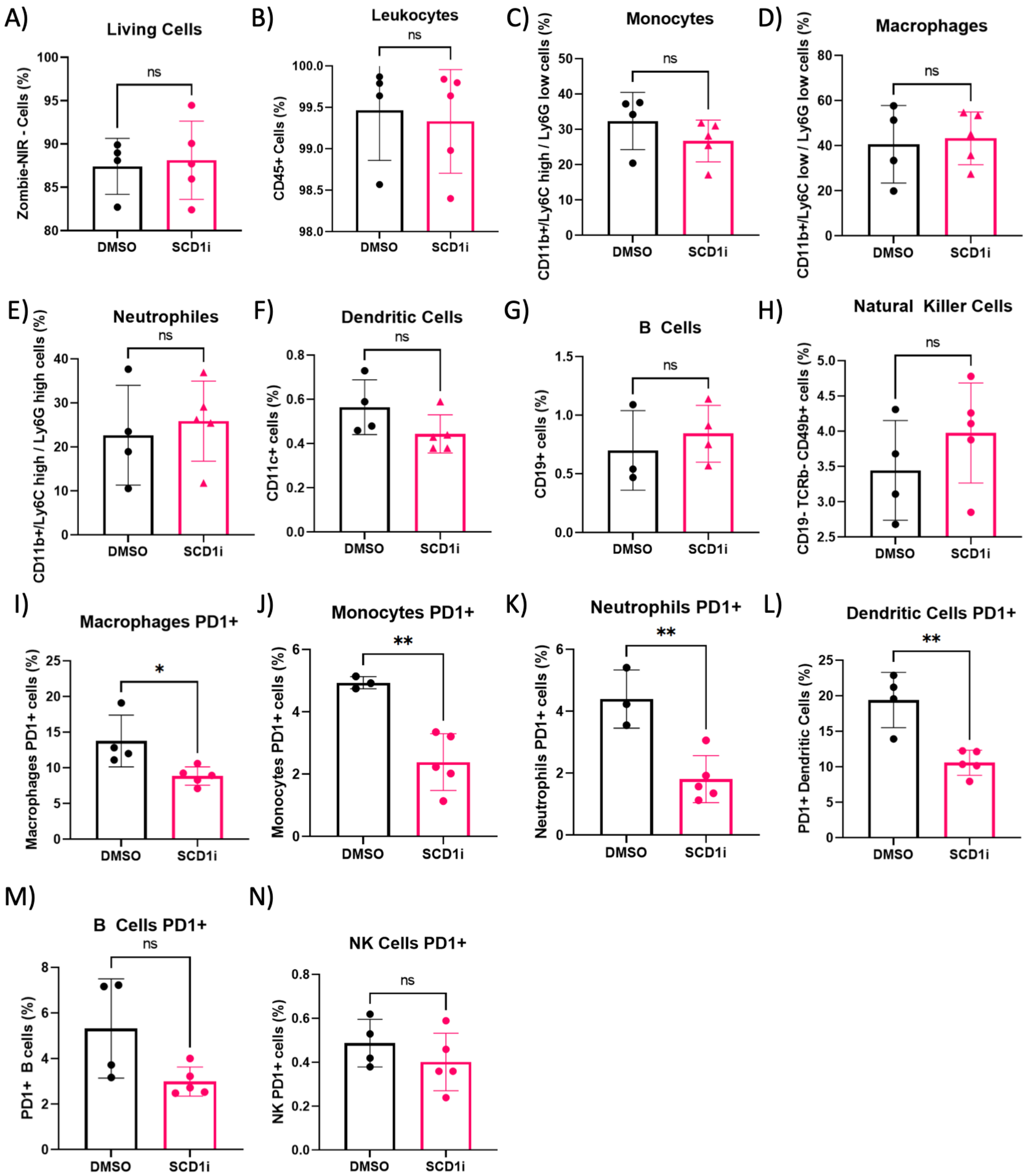

Supplementary Figure 6. SCD1 inhibition reduces PD-1 expression on myeloid cells in splenocytes.

A) Identification of live splenocytes incubated with conditioned media from E0771 cells treated with vehicle (DMSO) or the SCD1 inhibitor (SCD1i) for 24 h. B–H) Quantification of the proportions of leukocytes (B), monocytes (C), macrophages (D), neutrophils (E), dendritic cells (F), B cells (G), and natural killer (NK) cells (H). I–M) PD-1 expression on macrophages (I), monocytes (J), neutrophils (K), dendritic cells (L), and B cells (M). Data are presented as mean ± SD. Statistical significance was determined by one-way ANOVA followed by the appropriate post hoc multiple-comparison test. *P<0.05; **P<0.01

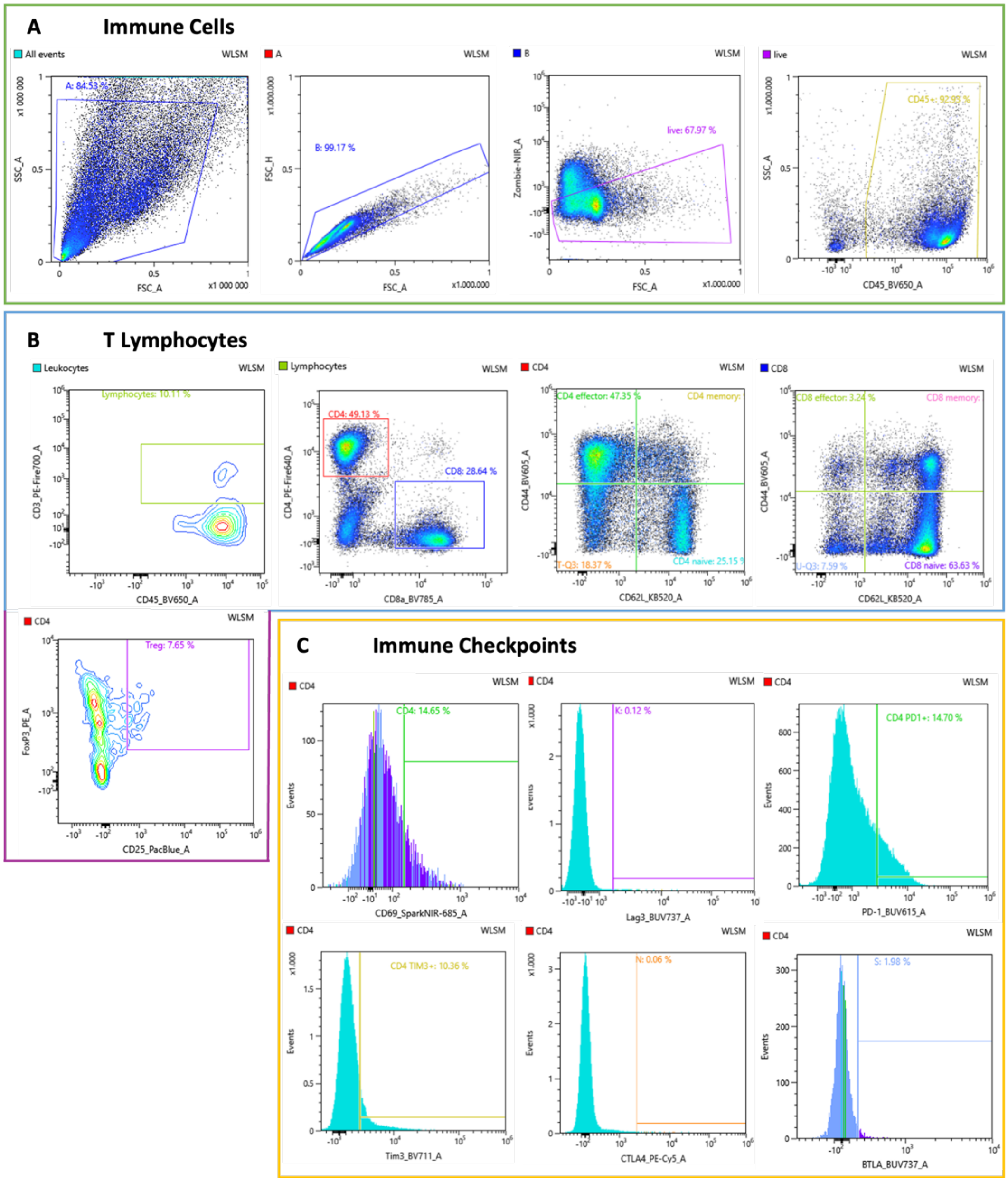

Supplementary Figure 7. **Flow Cytometry Gating of Lymphoid splenocytes.**

A) Cells were first gated for morphology (FSC-A/SSC-A) and single cells (FSC-A/FSC-H). Cells were then labeled with Zombie-Nir for cell viability. The cells were then labeled with a CD45 marker for leukocytes. B) T cells were gated from CD45+ cells and were CD3+. T cells were differentiated into Helper (CD4+) and Cytotoxic CD8+) T Cells. These two populations were differentiated into CD4+ or CD8+ effector (CD44+/CD62L-), memory (CD44+/CD62L+), and naïve (CD44-/CD62L+) T lymphocytes. CD4+ Cells were differentiated into regulator T lymphocytes (FoXP3+/CD25+) C) Cells were also labeled with markers for CD69 PD1, LAG-3, Tim-3, CTLA-4 and BTLA.

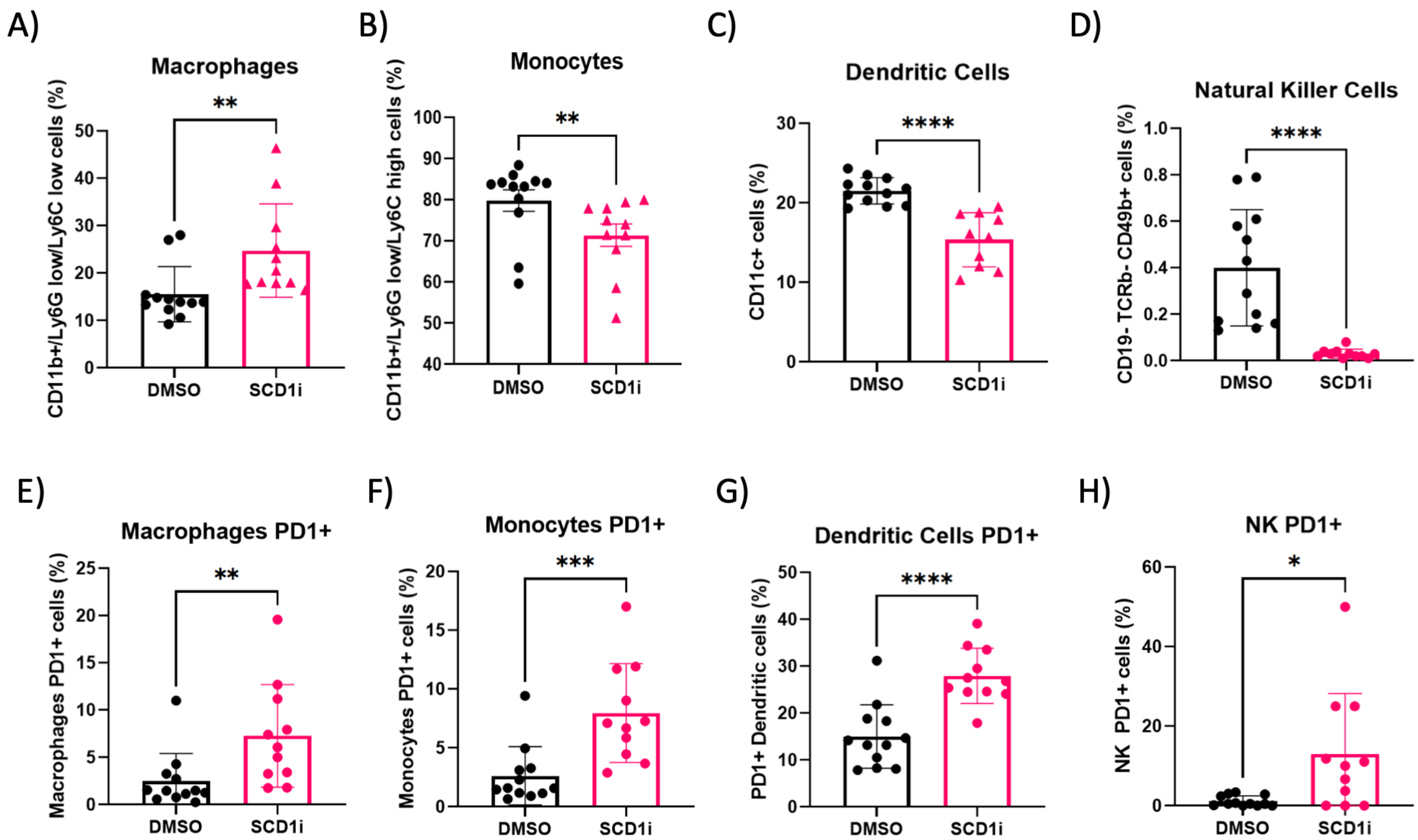

Supplementary Figure 8. SCD1 inhibition in vivo increases PD-1 expression on myeloid cells and NK cells in E0771 tumors.

A–D) Quantification of the proportions of macrophages (A), monocytes (B), dendritic cells (C), and natural killer (NK) cells (D) in E0771 tumors from mice treated with vehicle or SCD1 inhibitor for 7 days. E–H) Quantification of PD-1 expression on macrophages (E), monocytes (F), dendritic cells (G), and NK cells (H). Data are presented as mean ± SD. Statistical significance was determined by one-way ANOVA followed by the appropriate post hoc multiple-comparison test. *P < 0.05; **P < 0.01; ***P < 0.001.
